# Surveillance for Violent Deaths — National Violent Death Reporting System, 27 States, 2015

**DOI:** 10.15585/mmwr.ss6711a1

**Published:** 2018-09-28

**Authors:** Shane P.D. Jack, Emiko Petrosky, Bridget H. Lyons, Janet M. Blair, Allison M. Ertl, Kameron J. Sheats, Carter J. Betz

**Affiliations:** 1 Division of Violence Prevention, National Center for Injury Prevention and Control, CDC

## Abstract

**Problem/Condition:**

In 2015, approximately 62,000 persons died in the United States as a result of violence-related injuries. This report summarizes data from CDC’s National Violent Death Reporting System (NVDRS) regarding violent deaths from 27 U.S. states for 2015. Results are reported by sex, age group, race/ethnicity, location of injury, method of injury, circumstances of injury, and other selected characteristics.

**Reporting Period:**

2015.

**Description of System:**

NVDRS collects data regarding violent deaths obtained from death certificates, coroner/medical examiner reports, law enforcement reports, and secondary sources (e.g., child fatality review team data, supplemental homicide reports, hospital data, and crime laboratory data). This report includes data from 27 states that collected statewide data for 2015 (Alaska, Arizona, Colorado, Connecticut, Georgia, Hawaii, Kansas, Kentucky, Maine, Maryland, Massachusetts, Michigan, Minnesota, New Hampshire, New Jersey, New Mexico, New York, North Carolina, Ohio, Oklahoma, Oregon, Rhode Island, South Carolina, Utah, Vermont, Virginia, and Wisconsin). NVDRS collates documents for each death and links deaths that are related (e.g., multiple homicides, a homicide followed by a suicide, or multiple suicides) into a single incident.

**Results:**

For 2015, NVDRS captured 30,628 fatal incidents involving 31,415 deaths in the 27 states included in this report. The majority (65.1%) of deaths were suicides, followed by homicides (23.5%), deaths of undetermined intent (9.5%), legal intervention deaths (1.3%) (i.e., deaths caused by law enforcement and other persons with legal authority to use deadly force, excluding legal executions), and unintentional firearm deaths (<1.0%). (The term “legal intervention” is a classification incorporated into the *International Classification of Diseases, Tenth Revision* [*ICD-10*] and does not denote the lawfulness or legality of the circumstances surrounding a death caused by law enforcement.) Demographic patterns varied by manner of death. Suicide rates were highest among males, non-Hispanic American Indian/Alaska Natives, non-Hispanic whites, adults aged 45–54 years, and men aged ≥75 years. The most common method of injury was a firearm. Suicides often were preceded by a mental health, intimate partner, substance abuse, or physical health problem, or a crisis during the previous or upcoming 2 weeks. Homicide rates were higher among males and persons aged <1 year and 20–34 years. Among males, non-Hispanic blacks accounted for the majority of homicides and had the highest rate of any racial/ethnic group. Homicides primarily involved a firearm, were precipitated by arguments and interpersonal conflicts, were related to intimate partner violence (particularly for females), or occurred in conjunction with another crime. When the relationship between a homicide victim and a suspected perpetrator was known, an acquaintance/friend or an intimate partner frequently was involved. Legal intervention death rates were highest among males and persons aged 20–54 years; rates among non-Hispanic black males were approximately double the rates of those among non-Hispanic white males. Precipitating circumstances for legal intervention deaths most frequently were an alleged criminal activity in progress, the victim reportedly using a weapon in the incident, a mental health or substance abuse problem (other than alcohol abuse), an argument or conflict, or a recent crisis (during the previous or upcoming 2 weeks). Unintentional firearm deaths were more frequent among males, non-Hispanic whites, and persons aged 10–24 years; these deaths most often occurred while the shooter was playing with a firearm and most often were precipitated by a person unintentionally pulling the trigger or mistakenly thinking the firearm was unloaded. Deaths of undetermined intent were more frequent among males, particularly non-Hispanic black and American Indian/Alaska Native males, and persons aged 30–54 years. Substance abuse, mental health problems, physical health problems, and a recent crisis were the most common circumstances preceding deaths of undetermined intent. In 2015, approximately 3,000 current or former military personnel died by suicide. The majority of these decedents were male, non-Hispanic white, and aged 45–74 years. Most suicides among military personnel involved a firearm and were precipitated by mental health, physical health, and intimate partner problems, as well as a recent crisis.

**Interpretation:**

This report provides a detailed summary of data from NVDRS for 2015. The results indicate that deaths resulting from self-inflicted or interpersonal violence most frequently affect males and certain age groups and minority populations. Mental health problems, intimate partner problems, interpersonal conflicts, and general life stressors were primary precipitating events for multiple types of violent deaths, including suicides among current or former military personnel.

**Public Health Action:**

NVDRS data are used to monitor the occurrence of violence-related fatal injuries and assist public health authorities in the development, implementation, and evaluation of programs and policies to reduce and prevent violent deaths. For example, Virginia VDRS data are used to help identify suicide risk factors among active duty service members, Oregon VDRS suicide data are used to coordinate information and activities across community agencies that support veterans and active duty service members, and Arizona VDRS data are used to develop recommendations for primary care providers who deliver care to veterans. The continued development and expansion of NVDRS to include all 50 states, U.S. territories, and the District of Columbia are essential to public health efforts to reduce deaths due to violence.

## Introduction

In 2015, approximately 62,000 deaths in the United States were attributed to violence-related injuries (*1*). Suicide was the 10th leading cause of death overall in the United States and disproportionately affected young and middle-aged populations. Suicide was among the top two leading causes of death for persons aged 15–34 years and among the top four for persons aged 35–54 years. Non-Hispanic American Indian/Alaska Native and non-Hispanic white males were disproportionately affected by suicide.

Homicide was the 16th leading cause of death overall in the United States but disproportionately affected young persons (*1*). Homicide was the third leading cause of death for children aged 1–4 years and persons aged 15–34 years and the fourth leading cause of death for children aged 5–14 years. Young non-Hispanic black males were disproportionately affected by homicide, which was the leading cause of death among non-Hispanic black males aged 15–34 years.

Public health authorities require accurate, timely, and comprehensive surveillance data to better understand and ultimately prevent the occurrence of violent deaths in the United States (*2*). In 2000, in response to an Institute of Medicine* report noting the need for a national fatal intentional injury surveillance system (*3*), CDC began planning to implement the National Violent Death Reporting System (NVDRS) (*2*). The goals of NVDRS are to

- collect and analyze timely, high-quality data for monitoring the magnitude and characteristics of violent deaths at national, state, and local levels;
- ensure data are disseminated routinely and expeditiously to public health officials, law enforcement officials, policymakers, and the public;
- ensure data are used to develop, implement, and evaluate programs and strategies that are intended to reduce and prevent violent deaths and injuries at national, state, and local levels; and
- build and strengthen partnerships among organizations and communities at national, state, and local levels to ensure that data are collected and used to reduce and prevent violent deaths and injuries.

NVDRS is a state-based active surveillance system that collects data on the characteristics and circumstances associated with all violence-related deaths in participating states. Deaths include homicides, suicides, legal intervention deaths (i.e., deaths caused by law enforcement acting in the line of duty and other persons with legal authority to use deadly force but excluding legal executions), unintentional firearm deaths, and deaths of undetermined intent.^†^ (The term “legal intervention” is a classification incorporated into the *International Classification of Diseases, Tenth Revision* [*ICD-10*] and does not denote the lawfulness or legality of the circumstances surrounding a death caused by law enforcement.) NVDRS data are used to assist the development, implementation, and evaluation of programs and strategies designed to reduce and prevent violent deaths at the national, state, and local levels.

Before implementation of NVDRS, single data sources (e.g., death certificates or law enforcement reports) provided only limited information and few circumstances from which to understand patterns of violent deaths. NVDRS fills this surveillance gap by providing more detailed information. NVDRS is the first system to 1) provide detailed information on circumstances precipitating violent deaths, 2) link multiple source documents so that each incident can contribute to the study of patterns of violent deaths, and 3) link multiple deaths that are related to one another (e.g., multiple homicides, suicide pacts, or homicide followed by suicide of the suspected perpetrator).

NVDRS data collection began in 2003 with six participating states (Maryland, Massachusetts, New Jersey, Oregon, South Carolina, and Virginia). Seven states (Alaska, Colorado, Georgia, North Carolina, Oklahoma, Rhode Island, and Wisconsin) began data collection in 2004, four (California, Kentucky, New Mexico, and Utah) in 2005, two (Ohio and Michigan) in 2010, and 14 (Arizona, Connecticut, Hawaii, Iowa, Illinois, Indiana, Kansas, Maine, Minnesota, New Hampshire, New York, Pennsylvania, Vermont, and Washington) in 2015. Eight states (Alabama, California, Delaware, Louisiana, Missouri, Nebraska, Nevada, and West Virginia), the District of Columbia, and Puerto Rico began data collection in 2017 (Figure). CDC provides funding for state participation, and the ultimate goal is for NVDRS to expand to include all 50 states, U.S. territories, and the District of Columbia.^§^

**FIGURE:**
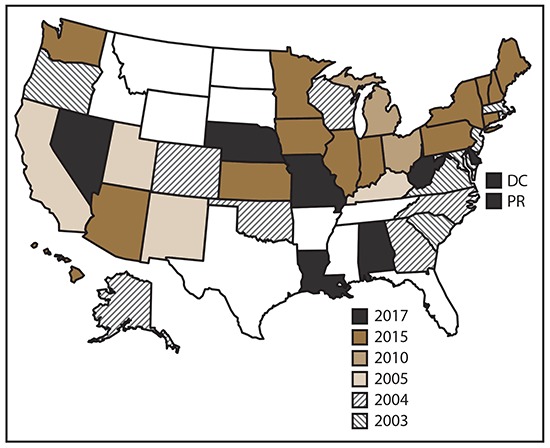
States participating in the National Violent Death Reporting System, by year of initial data collection,* United States and Puerto Rico, 2003–2017 **Abbreviations:** DC = District of Columbia; PR = Puerto Rico. * California began collecting data for a subset of violent deaths in 2005 but ended data collection in 2009. In 2017, California resumed data collection. Michigan collected data for a subset of violent deaths during 2010–2013 and collected statewide data beginning in 2014. Illinois, Indiana, Iowa, Pennsylvania, and Washington began collecting data for a subset of violent deaths in 2015. Alabama, California, Louisiana, Missouri, and Nebraska began collecting data for a subset of violent deaths in 2017.

In 2015, CDC collected data from 32 states. Five states (Iowa, Illinois, Indiana, Pennsylvania, and Washington) conducted a pilot in their first year of funding in 2015; thus their data are not included in this report. This report summarizes data from 27 states that collected information on all violent deaths occurring in their state in 2015 (Alaska, Arizona, Colorado, Connecticut, Georgia, Hawaii, Kansas, Kentucky, Maine, Maryland, Massachusetts, Michigan, Minnesota, New Hampshire, New Jersey, New Mexico, New York, North Carolina, Ohio, Oklahoma, Oregon, Rhode Island, South Carolina, Utah, Vermont, Virginia, and Wisconsin); these states account for approximately 46.9% of the U.S. population (*1*,*4*). Suicides among current or former military personnel also are highlighted in this report. NVDRS data are updated annually and are available to the public through CDC’s Web-based Injury Statistics Query and Reporting System (WISQARS)^¶^ at https://www.cdc.gov/injury/wisqars/nvdrs.html. Case-level NVDRS data are available to applicants who meet eligibility requirements via access to the NVDRS Restricted Access Database (https://www.cdc.gov/ViolencePrevention/NVDRS/RAD.html)*.*

## Methods

NVDRS compiles information from multiple data sources. The three required data sources are death certificates, coroner/medical examiner reports, and law enforcement reports. Certain participating states also collect information from secondary sources (e.g., child fatality review team data, supplemental homicide reports, and crime laboratory data). NVDRS collates documents for each death and links deaths that are related (e.g., multiple homicides, a homicide followed by a suicide, or multiple suicides) into a single incident. The ability to analyze linked data permits comprehensive assessment of violent deaths. This report presents selected data for 2015. Additional data from 2015 are available (Supplementary Tables, https://stacks.cdc.gov/view/cdc/56878).

In NVDRS, a violent death is defined as a death resulting from the intentional use of physical force or power, threatened or actual, against oneself, another person, or a group or community (*5*). Information is also collected about unintentional firearm deaths (i.e., a death resulting from a penetrating injury or gunshot wound from a weapon that uses a powder charge to fire a projectile when there was a preponderance of evidence that the shooting was not intentionally directed at the victim) and deaths of undetermined intent (i.e., a death that results from the use of force or power against oneself or another person for which the evidence indicating one manner of death is no more compelling than evidence indicating another). NVDRS cases are coded on the basis of *ICD-10 *(*6*) or the manner of death assigned by the coroner/medical examiner or law enforcement. Cases are included if they are assigned *ICD-10* codes (Box 1) or if the manner of death specified in at least one of the three primary data sources is consistent with NVDRS case definitions.

BOX 1 ***International Classification of Diseases, Tenth Revision (ICD-10)* codes used in the National Violent Death Reporting System**

**Table:** 

Manner of death	Death ≤1 year after injury	Death >1 year after injury	Death any time after injury
Intentional self-harm (suicide)	X60–X84	Y87.0	U03 (attributable to terrorism)
Assault (homicide)	X85–X99, Y00–Y09	Y87.1	U01, U02 (attributable to terrorism)
Event of undetermined intent	Y10–Y34	Y87.2, Y89.9	N/A
Unintentional exposure to inanimate mechanical forces (firearms)	W32–W34	Y86	N/A
Legal intervention (excluding executions, Y35.5)	Y35.0–Y35.4, Y35.6, Y35.7	Y89.0	N/A

Variables analyzed in NVDRS include

- manner of death (i.e., the intent [homicide/legal intervention, suicide, unintentional, undetermined] of the person inflicting a fatal injury);
- mechanism of injury (i.e., the method used to inflict a fatal injury) (Box 2);
  BOX 2 **Methods used to inflict injury — National Violent Death Reporting System, 27 states, 2015**
  - Firearm: method that uses a powder charge to fire a projectile
  - Hanging/strangulation/suffocation: hanging by the neck, manual strangulation, or plastic bag over the head
  - Poisoning: street drug, alcohol, pharmaceutical, carbon monoxide, gas, rat poison, or insecticide
  - Sharp instrument: knife, razor, machete, or pointed instrument (e.g., chisel or broken glass)
  - Blunt instrument: club, bat, rock, or brick
  - Fall: being pushed or jumping
  - Motor vehicle: (e.g., car, bus, motorcycle, or other transport vehicle)
  - Personal weapons: (e.g., hands, fists, or feet)
  - Drowning: inhalation of liquid in bathtub, lake, or other source of water/liquid
  - Fire/burns: inhalation of smoke or the direct effects of fire or chemical burns
  - Intentional neglect: starvation, lack of adequate supervision, or withholding of health care
  - Other: any method other than those already listed
  - Unknown: method not reported or not known
- toxicology findings (i.e., for decedents who were tested);
- circumstances preceding injury (i.e., the events that preceded and were identified by investigators as relevant and therefore might have contributed to the infliction of a fatal injury) (Box 3);
  BOX 3 **Circumstances preceding fatal injury, by manner of death — National Violent Death Reporting System, 27 states, 2015**
  **Suicide/Undetermined Intent**
  - Intimate partner problem: decedent was experiencing problems with a current or former intimate partner.
  - Suicide of friend or family: decedent was distraught over, or reacting to, the suicide of a friend or family member.
  - Other death of friend or family: decedent was distraught over, or reacting to, the recent nonsuicide death of a friend or family member.
  - Physical health problem: decedent was experiencing physical health problems (e.g., a recent cancer diagnosis, chronic pain).
  - Job problem: decedent was either experiencing a problem at work or was having a problem with joblessness.
  - Recent criminal legal problem: decedent was facing criminal legal problems.
  - Non-criminal legal problem: decedent was facing civil legal problems (e.g., a child custody, civil lawsuit).
  - Financial problem: decedent was experiencing problems such as bankruptcy, overwhelming debt, or foreclosure of a home or business.
  - Eviction or loss of home: decedent was experiencing a recent eviction or other loss of housing.
  - School problem: decedent was experiencing a problem such as poor grades, bullying, social exclusion at school, or performance pressures.
  - Traumatic anniversary: the incident occurred on or near the anniversary of a traumatic event in the decedent’s life.
  - Exposure to disaster: decedent was exposed to a disaster (e.g., earthquake, bombing).
  - Left a suicide note: decedent left a note, e-mail message, video, or other communication indicating intent to die by suicide.
  - Disclosed intent to die by suicide: decedent had previously expressed suicidal feelings to another person with time for that person to intervene.
  - History of suicidal thoughts or plans: decedent had previously expressed suicidal thoughts or plans.
  - History of suicide attempts: decedent had previously attempted suicide before the fatal incident.
  **Homicide/Legal Intervention**
  - Jealousy (lovers’ triangle): jealousy or distress over an intimate partner’s relationship or suspected relationship with another person.
  - Stalking: pattern of unwanted harassing or threatening tactics by either the decedent or suspect.
  - Prostitution: prostitution or related activity that includes prostitutes, pimps, clients, or others involved in such activity.
  - Drug involvement: drug dealing, drug trade, or illegal drug use.
  - Brawl: mutual physical fight involving three or more persons.
  - Mercy killing: decedent wished to die because of terminal or hopeless disease or condition, and documentation indicates that the decedent wanted to be killed.
  - Victim was a bystander: decedent was not the intended target in the incident (e.g., pedestrian walking past a gang fight).
  - Victim was a police officer on duty: decedent was a law enforcement officer killed in the line of duty.
  - Victim was an intervener assisting a crime victim: decedent was attempting to assist a crime victim at the time of the incident (e.g., child attempts to intervene and is killed while trying to assist a parent who is being assaulted).
  - Victim used a weapon: decedent used a weapon to attack or defend during the course of the incident.
  - Intimate partner violence–related: incident is related to conflict between current or former intimate partners; includes the death of an intimate partner or nonintimate partners (e.g., child or parent) killed to cause pain to an intimate partner.
  - Hate crime: decedent was selected intentionally because of his or her actual or perceived gender, religion, sexual orientation, race/ethnicity, or disability.
  - Mentally ill suspect: suspect’s attack on decedent was believed to be the direct result of a mental illness.
  - Drive-by shooting: suspect drove near the decedent and fired a weapon while driving.
  - Walk-by assault: decedent was killed by a targeted attack (e.g., ambush) where the suspect fled on foot.
  - Random violence: decedent was killed by a random act of violence.
  - Gang-related: incident resulted from gang activity or gang rivalry; not used if the decedent was a gang member and the death did not appear to result from gang activity.
  **All Manners of Death (Except Unintentional Firearm)**
  - Current depressed mood: decedent was perceived by self or others to be depressed.
  - Current diagnosed mental health problem: decedent was identified as having a mental health disorder or syndrome listed in the *Diagnostic and Statistical Manual, Version IV* (*DSM-IV*), with the exception of alcohol and other substance dependence (these are captured in separate variables).
  - Type of mental health diagnosis: identifies the *DSM-IV* diagnosis made by a medical or mental health practitioner.
  - Current mental health treatment: decedent was currently receiving mental health treatment as evidenced by a current prescription for a psychotropic medication or visit to a mental health professional in the previous 2 months.
  - History of treatment for mental health problem: decedent was identified as having ever received mental health treatment during the decedent’s lifetime.
  - Alcohol/other substance problem: decedent was perceived by self or others to have a problem with, or to be addicted to, alcohol or other drugs.
  - Other addiction: decedent was perceived by self or others to have an addiction other than alcohol or other substance abuse (e.g. gambling, sex).
  - Family relationship problem: decedent was experiencing problems with a family member, other than an intimate partner.
  - Other relationship problem: decedent was experiencing problems with a friend or associate (other than an intimate partner or family member).
  - History of child abuse/neglect: decedent had history of physical, sexual, or psychological abuse; physical, emotional, or educational neglect; or exposure to a violent environment or inadequate supervision by a caretaker as a child.
  - Caretaker abuse/neglect led to death: decedent was experiencing physical, sexual, or psychological abuse; physical, emotional, or educational neglect; or exposure to a violent environment or inadequate supervision by a caretaker that led to death.
  - Perpetrator of interpersonal violence in previous month: decedent perpetrated interpersonal violence during the previous month.
  - Victim of interpersonal violence in previous month: decedent was the target of interpersonal violence during the past month.
  - Physical fight: a physical fight between two individuals that resulted in the death of the decedent who was either involved in the fight, a bystander, or trying to stop the fight.
  - Argument or conflict: a specific argument or disagreement occurred during the incident.
  - Precipitated by another crime: incident occurred as the result of another serious crime.
  - Nature of crime: identifies the specific type of other crime that occurred during the incident (e.g., robbery or drug trafficking).
  - Crime in progress: serious crime was in progress at the time of the incident.
  - Terrorist attack: decedent was injured in a terrorist attack, leading to death.
  - Crisis during previous or upcoming 2 weeks: current crisis or acute precipitating event(s) that either occurred in the previous 2 weeks or was impending in the following 2 weeks (e.g., a trial for a criminal offense begins the following week).
  - Other crisis: a crisis related to a death but not captured by any of the standard circumstances.
  **Unintentional Firearm Death**
  *Context of Injury*
  - Hunting: death occurred any time after leaving home for a hunting trip and before returning home from a hunting trip.
  - Target shooting: shooter was aiming for a target and unintentionally hit the decedent; can be at a shooting range or an informal backyard setting (e.g., teenagers shooting at signposts on a fence).
  - Loading/unloading gun: gun discharged when the shooter was loading/unloading ammunition.
  - Cleaning gun: shooter pulled trigger or gun discharged while cleaning, repairing, assembling or disassembling gun.
  - Showing gun to others: showing the gun to another person when the gun discharged or the trigger was pulled.
  - Playing with gun: shooter and one or more others were playing with a gun when it discharged.
  - Celebratory firing: shooter fired gun in celebratory manner (e.g., on New Year’s Eve night).
  - Other context of injury: shooting occurred during some context other than those already described.
  *Mechanism of Injury*
  - Unintentionally pulled trigger: shooter unintentionally pulled the trigger (e.g., while grabbing the gun or holding it too tightly).
  - Thought gun safety was engaged: shooter thought the safety was on and gun would not discharge.
  - Thought unloaded/magazine disengaged: shooter thought the gun was unloaded because the magazine was disengaged.
  - Thought gun was unloaded: shooter thought the gun was unloaded for other unspecified reason.
  - Bullet ricochet: bullet ricocheted from its intended target and struck the decedent.
  - Gun defect or malfunction: gun had a defect or malfunctioned as determined by a trained firearm examiner.
  - Gun fired while holstering: gun was being replaced or removed from holster/clothing.
  - Dropped gun: gun discharged when it was dropped or when something was dropped on it.
  - Gun fired while operating safety/lock: shooter unintentionally fired the gun while operating the safety lock.
  - Gun mistaken for toy: gun was mistaken for a toy and was fired without the user understanding the danger.
  - Other mechanism of injury: shooting occurred as the result of a mechanism not already described.
- whether the decedent was a victim (i.e., a person who died as a result of a violence-related injury) or both a suspect and a victim (i.e., a person believed to have inflicted a fatal injury on a victim who then was fatally injured, such as the perpetrator of a homicide-suicide);
- information about any known suspects (i.e., a person or persons believed to have inflicted a fatal injury on a victim);
- incident (i.e., an occurrence in which one or more persons sustained a fatal injury that was linked to a common event or perpetrated by the same suspect during a 24-hour period); and
- type of incident (i.e., a combination of the manner of death and the number of victims in an incident).

NVDRS is an incident-based system, and all decedents associated with a given incident are grouped in one record. Decisions about whether two or more deaths are related and belong to the same incident are made on the basis of the timing of the injuries rather than on the timing of the deaths. Deaths resulting from injuries that occur within 24 hours of each other and are clearly linked by source documents (discussed under Manner of Death) would be considered part of the same incident. Examples of an incident include 1) a single isolated violent death, 2) two or more related homicides (including legal intervention deaths) when the fatal injuries were inflicted <24 hours apart, 3) two or more related suicides or deaths of undetermined intent when the fatal injuries were inflicted <24 hours apart, and 4) a homicide followed by a suicide when both fatal injuries were inflicted <24 hours apart (*7*).

Information collected from each data source is entered into the NVDRS web-based data entry system (*2*). This system simplifies data abstraction by allowing abstractors to enter data from multiple sources into the same incident record. Internal validation checks, hover-over features that define selected fields, and other quality control measures are included. Primacy rules and hierarchal algorithms related to the source documents occur at the state level. CDC provides access to the web-based system to each state, district, and territory. Project personnel are provided ongoing coding training to help increase data quality. Data are transmitted continuously via the web to a CDC-based server. No personally identifiable information is transmitted to CDC.

### Manner of Death

A manner (i.e., intent) of death for each decedent is assigned by a trained abstractor who integrates information from all source documents. The abstractor-assigned manner of death must agree with at least one required data source; typically, all source documents are consistent regarding the manner of death. When there is a discrepancy, the abstractor must assign a manner of death on the basis of the preponderance of evidence in the source documents, but such occurrences are rare (*7*). For example, if two sources report a death as a suicide and a third reports it as a death of undetermined intent, the death is coded as a suicide.

NVDRS data are categorized into five abstractor-assigned manners of death: 1) suicide, 2) homicide, 3) unintentional firearm, 4) undetermined intent, and 5) legal intervention.

- **Suicide.** Suicide is a death resulting from the use of force against oneself when a preponderance of evidence indicates that the use of force was intentional. This category also includes the following scenarios: 1) deaths of persons who intended only to injure rather than kill themselves; 2) persons who initially intended to kill themselves, changed their minds, but died as a result of the acts; 3) deaths associated with risk-taking behavior without clear intent to inflict fatal self-injury but associated with high risk for death (e.g., playing Russian roulette); 4) suicides that occurred while under the influence of substances or drugs, taken voluntarily; 5) suicides that occurred while under the influence of a mental illness; and 6) suicides involving another person providing only passive assistance to the decedent (e.g., supplying the means or information needed to complete the act). This category does not include deaths caused by chronic or acute substance abuse without the intent to die or deaths attributed to autoerotic behavior (e.g., self-strangulation during sexual activity). Corresponding *ICD-10* codes included in NVDRS are X60–X84, Y87.0, and U03 (Box 1).
- **Homicide.** Homicide is a death resulting from the use of physical force or power, threatened or actual, against another person, group, or community when a preponderance of evidence indicates that the use of force was intentional. Two special scenarios that CDC’s National Center for Health Statistics (NCHS) regards as homicides are included in the NVDRS case definition: 1) arson with no specified intent to injure someone and 2) a stabbing with intent unspecified. This category also includes the following scenarios: 1) deaths when the suspect intended to only injure rather than kill the victim, 2) deaths resulting from heart attack induced when the suspect uses force or power against the victim, 3) deaths that occur when a person kills an attacker in self-defense, 4) deaths resulting from a weapon that discharges unintentionally while being used to control or frighten a victim, 5) deaths attributed to child abuse without intent being specified, 6) deaths attributed to intentional act of neglect by one person against another, 7) deaths of a child after birth that results from a direct injury due to violence sustained before birth, and 8) deaths identified as a justifiable homicide where the person committing homicide was not a law enforcement officer. This category excludes vehicular homicide without intent to injure, unintentional poisoning deaths due to illegal or prescription drug overdose even when the person who provided drugs was charged with homicide, unintentional firearm deaths (a separate category in NVDRS), combat deaths or acts of war, deaths of unborn fetuses, and deaths of children after birth that resulted indirectly from violence sustained by the mother before birth (e.g., death from prematurity following premature labor brought on by violence). Corresponding *ICD-10* codes included in NVDRS are X85–X99, Y00–Y09, Y87.1, and U01–U02 (Box 1).
- **Unintentional firearm**. An unintentional firearm death is a death resulting from a penetrating injury or gunshot wound from a weapon that uses a powder charge to fire a projectile and for which a preponderance of evidence indicates that the shooting was not directed intentionally at the decedent. Examples include the following: 1) a person who dies as a result of a celebratory firing that was not intended to frighten, control, or harm anyone; 2) a person who unintentionally shoots himself when using a firearm to frighten, control, or harm another person; 3) a soldier who is shot during a field exercise but not in a combat situation; 4) a person who received a self-inflicted wound while playing with a firearm; 5) a person who mistakenly believes a gun is unloaded and shoots another person; 6) a child aged <6 years who shoots himself or another person; and 7) a child who dies after birth from an unintentional firearm injury that was sustained in utero. This category excludes injuries caused by unintentionally striking a person with the firearm (e.g., hitting a person on the head with the firearm rather than firing a projectile) and unintentional injuries from nonpowder guns (e.g., BB, pellet, or other compressed air-powered or gas-powered guns). Corresponding *ICD-10* codes included in NVDRS are W32–W34 and Y86 (Box 1).
- **Undetermined intent.** A death of undetermined intent is a death resulting from the use of force or power against oneself or another person for which the evidence indicating one manner of death is no more compelling than evidence indicating another. This category includes coroner/medical examiner rulings (e.g., accident or suicide, undetermined, jumped or fell, or self-inflicted injury) when records give no evidence or opinions in favor of either unintentional or intentional injury. Corresponding *ICD-10* codes included in NVDRS are Y10–Y34, Y87.2, and Y89.9 (Box 1).
- **Legal intervention.** A death from legal intervention is a death in which a person is killed or died as a result of a law enforcement officer or other peace officer (i.e., a person with specified legal authority to use deadly force), including military police, while on duty. (The term “legal intervention” is a classification from *ICD-10* [Y-35.0] and does not denote the lawfulness or legality of the circumstances surrounding the death.) Legal intervention deaths also include a small subset in which force was applied without clear lethal intent (e.g., during restraint or when applying force with a typically nondeadly weapon, such as a Taser, or in which the death occurred while the person was fleeing capture. This category excludes legal executions. Corresponding *ICD-10* codes included in NVDRS are Y35.0–Y35.4, Y35.6, Y35.7, and Y89.0 (Box 1).

### Variables Analyzed

NVDRS collects approximately 600 unique variables for each death. The number of variables recorded for each incident depends on the content and completeness of the source documents. Variables include manner of death; demographic information; *ICD-10* cause of death codes and text descriptors; location, date, and time of injury and death; toxicology results; bodily injuries; precipitating circumstances; victim-suspect relationship; and method of injury (Boxes 1, 2, and 3). NVDRS also has the capacity to collect information on specific populations, including military personnel. Military status is assessed from a variable originating from the death certificate of whether a decedent ever served in the U.S. Armed Forces. Decedents either were actively serving or had previously served in the military at time of death.

### Circumstances Preceding Death

Circumstances preceding death are defined as the precipitating events that contributed to the infliction of a fatal injury (Box 3). The circumstances are reported on the basis of the content of the coroner/medical examiner and law enforcement investigative reports. Certain circumstances are coded to a specific manner of death (e.g., suicide or death of undetermined intent); other circumstances are coded across all manners of death. The data abstractor selects from a list of potential circumstances and is required to code all circumstances that are known to relate to each incident. If circumstances are not known (e.g., for a body found in the woods with no other details reported), the data abstractor leaves the circumstances known variable blank; these deaths are excluded from the denominator for circumstance values. If either the coroner/medical examiner record or law enforcement report indicates the presence of a circumstance, then the abstractor endorses the circumstance (e.g., if the law enforcement report indicated that a decedent had disclosed an intent to die by suicide, then suicidal intent is endorsed).

### Coding Training and Quality Control

Ongoing coding support for data abstractors is provided through an e-mail help desk, monthly conference calls, annual in-person meetings with all states, and regular conference calls with individual states. States also can conduct additional abstractor training workshops and activities at their own discretion. An NVDRS coding manual (*7*) with CDC-issued standard guidance on coding criteria and examples for each data element is provided. Software features to enhance coding reliability include automated validation rules and a hover-over feature containing variable-specific information.

States are requested to perform annual blind reabstractions of a subset of cases using multiple abstractors to identify inconsistencies. CDC also runs a quality control analysis in which multiple variables are reviewed for their appropriateness, with special focus on abstractor-assigned variables (e.g., method and manner of death). If CDC finds inconsistencies, the state is notified and asked for a response or correction.

### Time Frame

States are required to report all deaths within 6 months of the end of each calendar year for the preceding January–December. States then have an additional 12 months to complete each incident record. Although states typically meet these requirements, additional details sometimes arrive after a deadline has passed. New incidents also might be identified after the deadline (e.g., a death certificate is revised, new evidence is obtained that changes a manner of death, or an *ICD-10* miscoding is corrected to meet NVDRS inclusion criteria). These additional data are incorporated into NVDRS. Analysis files are updated in real time in the web-based system. On the basis of a recent examination of the past 10 data years, CDC estimates that case counts are not likely to increase more than 1.0% after the 18-month data collection period.

### Fatal Injuries in 2015

This report provides data concerning fatal injuries meeting the NVDRS case definition for violent deaths in 2015 that were received by CDC as of September 28, 2017. The 27 participating states used vital statistics death certificate files or coroner/medical examiner reports to identify violent deaths meeting NVDRS case definitions. Each state reported all violent deaths of residents that occurred within the state and those of nonresidents for whom a fatal injury occurred within the state (i.e., occurrent deaths). When a violent death was identified, NVDRS data abstractors linked source documents, linked deaths within each incident, coded data elements, and wrote short narratives of the incident. State-level data were then consolidated and analyzed.

Numbers, percentages, and crude rates are presented in aggregate for all deaths by abstractor-assigned manner of death. Rates for cells with frequency <20 are not reported because of the instability of those rates (*8*). Rates could not be calculated for certain variables (e.g., precipitating circumstances) because denominators were unknown. Bridged-race 2015 population estimates were used as denominators in the crude rate calculations (*9*). For compatible numerators for rate calculations to be derived, records listing multiple races were recoded to a single race, when possible, using race-bridging methods described by NCHS (available at https://www.cdc.gov/nchs/nvss/bridged_race.htm).

## Results

### All Deaths Captured by NVDRS

#### Deaths by Manner

The 27 NVDRS states included in this report collected data concerning 30,628 incidents and 31,415 deaths that occurred in 2015. The crude death rate was 20.9 per 100,000 population. Suicides (n = 20,446; 65.1%) accounted for the highest rate of violent deaths (13.6 per 100,000 population), followed by homicides (n = 7,374; 23.5%) (4.9 per 100,000 population). Deaths of undetermined intent (n = 2,974; 9.5%), legal intervention deaths (n = 417; 1.3%), and unintentional firearm deaths (n = 204; <1.0%) occurred at 2.0, 0.3, and 0.1 per 100,000 population, respectively.

### Suicides

#### Sex, Race/Ethnicity, and Age Group

The 27 NVDRS states included in this report collected data for 2015 concerning 20,411 suicide incidents, which included 20,446 deaths (Table 1). Overall, the crude suicide rate was 13.6 per 100,000 population. The overall rate for males was nearly three and one-half times the rate for females (21.2 and 6.2 per 100,000 population, respectively); however, rates for males ranged from two to approximately nine times the rates for females across age groups and two to 4.5 times the rates for females across racial/ethnic groups. Adults aged 45–54 years and 55–64 years (20.2 and 18.3 per 100,000 population, respectively) had the highest rates of suicides across age groups. Youths aged 10–19 years accounted for <6.0% of all suicides and had the lowest rates among all age groups. Non-Hispanic whites accounted for the majority (83.6%) of suicides. Non-Hispanic American Indian/Alaska Natives had the highest rate of suicides (22.2 per 100,000 population).

**TABLE 1:** Number, percentage,* and rate^†^ of suicides, by decedent’s sex, age group, race/ethnicity, method used, and location in which injury occurred — National Violent Death Reporting System, 27 states,^§^ 2015

Characteristic	Male		Female		Total	
	No. (%)	Rate	No. (%)	Rate	No. (%)	Rate
**Age group (yrs)**						
<10	—^¶^	—^¶^	—^¶^	—^¶^	—**^¶^**	**—^¶^**
10–14	128 (<1.0)	2.6	62 (1.3)	1.3	**190 (<1.0)**	**2.0**
15–19	726 (4.6)	14.3	250 (5.3)	5.2	**976 (4.8)**	**9.9**
20–24	1,357 (8.6)	24.9	281 (5.9)	5.4	**1,638 (8.0)**	**15.4**
25–29	1,312 (8.4)	25.0	309 (6.5)	6.1	**1,621 (7.9)**	**15.7**
30–34	1,268 (8.1)	25.3	371 (7.8)	7.4	**1,639 (8.0)**	**16.4**
35–44	2,402 (15.3)	25.7	794 (16.7)	8.3	**3,196 (15.6)**	**16.9**
45–54	3,025 (19.3)	30.0	1,120 (23.6)	10.7	**4,145 (20.3)**	**20.2**
55–64	2,644 (16.8)	28.1	929 (19.6)	9.2	**3,573 (17.5)**	**18.3**
65–74	1,524 (9.7)	24.9	386 (8.1)	5.5	**1,910 (9.3)**	**14.6**
75–84	932 (5.9)	33.3	165 (3.5)	4.4	**1,097 (5.4)**	**16.8**
≥85	384 (2.4)	38.0	77 (1.6)	3.9	**461 (2.3)**	**15.5**
Unknown	0 (0.0)	—**	0 (0.0)	—**	**0 (0.0)**	—**
**Race/Ethnicity**						
White, non-Hispanic	13,159 (83.8)	26.4	3,943 (83.1)	7.6	**17,102 (83.6)**	**16.9**
Black, non-Hispanic	984 (6.3)	9.9	244 (5.1)	2.2	**1,228 (6.0)**	**5.9**
American Indian/Alaska Native, non-Hispanic	282 (1.8)	33.8	96 (2.0)	11.1	**378 (1.8)**	**22.2**
Asian/Pacific Islander	384 (2.4)	9.8	192 (4.0)	4.5	**576 (2.8)**	**7.1**
Hispanic^††^	847 (5.4)	9.1	249 (5.2)	2.8	**1,096 (5.4)**	**6.0**
Other	36 (<1.0)	—**	16 (<1.0)	—**	**52 (<1.0)**	—**
Unknown	10 (<1.0)	—**	4 (<1.0)	—**	**14 (<1.0)**	—**
**Method**						
Firearm	8,551 (54.5)	11.6	1,358 (28.6)	1.8	**9,909 (48.5)**	**6.6**
Hanging/strangulation/suffocation	4,550 (29.0)	6.2	1,357 (28.6)	1.8	**5,907 (28.9)**	**3.9**
Poisoning	1,441 (9.2)	1.9	1,562 (32.9)	2.0	**3,003 (14.7)**	**2.0**
Fall	352 (2.2)	0.5	152 (3.2)	0.2	**504 (2.5)**	**0.3**
Sharp instrument	309 (2.0)	0.4	86 (1.8)	0.1	**395 (1.9)**	**0.3**
Motor vehicles (e.g., buses, motorcycles, other transport vehicles)	233 (1.5)	0.3	80 (1.7)	0.1	**313 (1.5)**	**0.2**
Drowning	146 (<1.0)	0.2	94 (2.0)	0.1	**240 (1.2)**	**0.2**
Fire/burns	64 (<1.0)	0.1	29 (<1.0)	0.0	**93 (<1.0)**	**0.1**
Blunt instrument	8 (<1.0)	—**	4 (<1.0)	—**	**12 (<1.0)**	—**
Intentional neglect	0 (0.0)	—**	0 (0.0)	—**	**0 (0.0)**	—**
Personal weapons (e.g., hands, feet, fists)	0 (0.0)	—**	0 (0.0)	—**	**0 (0.0)**	—**
Other (single method)	26 (<1.0)	—**	12 (<1.0)	—**	**38 (<1.0)**	—**
Unknown	22 (<1.0)	—**	10 (<1.0)	—**	**32 (<1.0)**	—**
**Location**						
House/apartment	11,412 (72.7)	15.4	3,747 (79.0)	4.9	**15,159 (74.1)**	**10.1**
Natural area	867 (5.5)	1.2	185 (3.9)	0.2	**1,052 (5.1)**	**0.7**
Motor vehicle	724 (4.6)	1.0	151 (3.2)	0.2	**875 (4.3)**	**0.6**
Street/highway	469 (3.0)	0.6	88 (1.9)	0.1	**557 (2.7)**	**0.4**
Hotel/motel	331 (2.1)	0.4	162 (3.4)	0.2	**493 (2.4)**	**0.3**
Parking lot/public garage/public transport	287 (1.8)	0.4	48 (1.0)	0.1	**335 (1.6)**	**0.2**
Other location^§§^	1,411 (9.0)	—**	284 (6.0)	—**	**1,695 (8.3)**	—**
Unknown	201 (1.3)	—**	79 (1.7)	—**	**280 (1.4)**	—**
**Total**	**15,702 (100)**	**21.2**	**4,744 (100)**	**6.2**	**20,446 (100)**	**13.6**

* Percentages might not total 100% due to rounding.

^†^ Per 100,000 population.

^§^ Alaska, Arizona, Colorado, Connecticut, Georgia, Hawaii, Kansas, Kentucky, Maine, Maryland, Massachusetts, Michigan, Minnesota, New Hampshire, New Jersey, New Mexico, New York, North Carolina, Ohio, Oklahoma, Oregon, Rhode Island, South Carolina, Utah, Vermont, Virginia, and Wisconsin.

^¶^ Suicide is not reported for decedents aged <10 years, as per standard in the suicide prevention literature.

** Rates not reported when number of decedents is <20 or when characteristic response is other or unknown.

^††^ Includes persons of any race.

^§§^ Includes park/playground/sports or athletic area, jail/prison, commercial/retail area, railroad tracks, hospital or medical facility, supervised residential facility, preschool/school/college/school bus, farm, industrial or construction area, office building, abandoned house/building/warehouse, bar/nightclub, and other unspecified location.

Among males, more than half (51.4%) of suicide decedents were aged 35–64 years. Men aged ≥85 years had the highest rate, followed by men aged 75–84 and 45–54 years (38.0, 33.3, and 30.0 per 100,000 population, respectively) (Table 1). Non-Hispanic American Indian/Alaska Natives had the highest rate of suicides (33.8 per 100,000 population), followed by non-Hispanic whites (26.4 per 100,000 population). These rates were approximately three times the rates for Asian/Pacific Islander (9.8 per 100,000 population) and Hispanic males (9.1 per 100,000 population). Among females, decedents aged 35–64 years also accounted for the majority (59.9%) of suicides. Women aged 45–54 years had the highest rate of suicide (10.7 per 100,000 population). Rates were highest for non-Hispanic American Indian/Alaska Native (11.1 per 100,000 population) and non-Hispanic white (7.6 per 100,000 population) females and lowest for non-Hispanic black (2.2 per 100,000 population) and Hispanic (2.8 per 100,000 population) females.

#### Method and Location of Injury

Firearms were used in nearly half (48.5%) of suicides, followed by hanging/strangulation/suffocation (28.9%) and poisoning (14.7%) (6.6, 3.9, and 2.0 per 100,000 population, respectively); the remaining methods used accounted for 7.9% of suicides (Table 1). Among males, the most common method used was a firearm (54.5%), followed by hanging/strangulation/suffocation (29.0%). Among females, poisoning (32.9%), a firearm (28.6%), and hanging/strangulation/suffocation (28.6%) were used in nearly equal proportions. The most common place of suicide was a house/apartment (74.1%) for both males and females (72.7% and 79.0%, respectively), followed by a natural area (5.1%), a motor vehicle (4.3%), a street/highway (2.7%), and a hotel/motel (2.4%).

#### Toxicology Results of Decedent

Tests for alcohol were conducted for 53.6% of suicide decedents (Table 2). Tests for amphetamines, antidepressants, benzodiazepines, cocaine, marijuana, and opioids were conducted for 37.2%, 26.5%, 39.7%, 39.0%, 32.1%, and 41.8% of decedents, respectively. Among those with positive results for alcohol (40.6%), 66.3% had blood alcohol concentration (BAC) ≥0.08 g/dL. Results for opioids (including illicit and prescription drugs) were positive in 26.6% of decedents tested for these substances. Results for amphetamines, cocaine, and marijuana were positive in 9.7%, 6.3%, and 22.4% of decedents tested, respectively. Of those tested for antidepressants, 40.8% had positive results at the time of their death, and 30.3% of those tested for benzodiazepines had positive results. Carbon monoxide was tested for in substantially fewer decedents (6.4%), but was identified in more than one third of those decedents (37.8%).

**TABLE 2:** Number* and percentage of suicide decedents who were tested for alcohol and drugs whose results were positive,^†^ by toxicology variable — National Violent Death Reporting System, 27 states,^§^ 2015

Toxicology variable	Tested	Positive
	No. (%)	No. (%)
BAC^¶^	10,950 (53.6)	4,442 (40.6)
Alcohol <0.08 g/dL		1,297 (29.2)
Alcohol ≥0.08 g/dL		2,943 (66.3)
Alcohol positive, level unknown		202 (4.5)
Amphetamines	7,615 (37.2)	736 (9.7)
Anticonvulsants	4,126 (20.2)	741 (18.0)
Antidepressants	5,425 (26.5)	2,214 (40.8)
Antipsychotics	4,312 (21.1)	482 (11.2)
Barbiturates	6,287 (30.7)	218 (3.5)
Benzodiazepines	8,124 (39.7)	2,464 (30.3)
Carbon monoxide	1,315 (6.4)	497 (37.8)
Cocaine	7,978 (39.0)	499 (6.3)
Marijuana	6,569 (32.1)	1,471 (22.4)
Muscle relaxants	4,293 (21.0)	345 (8.0)
Opioids	8,554 (41.8)	2,279 (26.6)
Other drugs/substances**	4,585 (22.4)	3,514 (76.6)

**Abbreviation:** BAC = blood alcohol concentration.

* N = 20,446.

^†^ Percentage is of decedents tested for toxicology variable.

^§^ Alaska, Arizona, Colorado, Connecticut, Georgia, Hawaii, Kansas, Kentucky, Maine, Maryland, Massachusetts, Michigan, Minnesota, New Hampshire, New Jersey, New Mexico, New York, North Carolina, Ohio, Oklahoma, Oregon, Rhode Island, South Carolina, Utah, Vermont, Virginia, and Wisconsin.

^¶^ BAC ≥0.08 g/dL is over the legal limit in all states and is used as the standard for intoxication.

** Other drugs/substances indicated if any results were positive; levels for these drugs/substances are not measured.

#### Precipitating Circumstances

Precipitating circumstances were known for 18,764 (91.8%) of suicide decedents (Table 3). Overall, mental health problems were the most common circumstance, with 37.5% of decedents described as experiencing a depressed mood at the time of their death, 50.1% as having a current diagnosed mental health problem, and 27.4% as currently receiving mental health treatment. Among the 9,399 decedents with a current diagnosed mental health problem, depression/dysthymia (75.3%), anxiety disorder (16.8%), and bipolar disorder (15.2%) were the most common diagnoses.

**TABLE 3:** Number* and percentage^†^ of suicides, by precipitating circumstances and decedent’s sex — National Violent Death Reporting System, 27 states,^§^ 2015

Precipitating circumstance	Male	Female	Total
	No. (%)	No. (%)	No. (%)
**Mental health/Substance abuse**			
Current diagnosed mental health problem^¶^	6,464 (45.1)	2,934 (66.2)	**9,398 (50.1)**
Depression/dysthymia	4,778 (73.9)	2,298 (78.3)	**7,076 (75.3)**
Anxiety disorder	993 (15.4)	586 (20.0)	**1,579 (16.8)**
Bipolar disorder	883 (13.7)	548 (18.7)	**1,431 (15.2)**
Schizophrenia	373 (5.8)	136 (4.6)	**509 (5.4)**
PTSD	336 (5.2)	88 (3.0)	**424 (4.5)**
ADD/ADHD	171 (2.6)	55 (1.9)	**226 (2.4)**
OCD	31 (<1.0)	14 (<1.0)	**45 (<1.0)**
Eating disorder	6 (<1.0)	18 (<1.0)	**24 (<1.0)**
Other	394 (6.1)	158 (5.4)	**552 (5.9)**
Unknown	543 (8.4)	217 (7.4)	**760 (8.1)**
Current depressed mood	5,318 (37.1)	1,720 (38.8)	**7,038 (37.5)**
History of ever being treated for a mental health problem	4,501 (31.4)	2,216 (50.0)	**6,717 (35.8)**
Current mental health treatment	3,319 (23.2)	1,822 (41.1)	**5,141 (27.4)**
Alcohol problem	2,633 (18.4)	635 (14.3)	**3,268 (17.4)**
Substance abuse problem (excludes alcohol)	2,291 (16.0)	793 (17.9)	**3,084 (16.4)**
Other addiction (e.g., gambling, sex)	102 (<1.0)	37 (<1.0)	**139 (<1.0)**
**Interpersonal**			
Intimate partner problem	4,082 (28.5)	1,016 (22.9)	**5,098 (27.2)**
Family relationship problem	1,174 (8.2)	497 (11.2)	**1,671 (8.9)**
Other death of family member or friend during past 5 years	813 (5.7)	368 (8.3)	**1,181 (6.3)**
Perpetrator of interpersonal violence during past month	376 (2.6)	38 (<1.0)	**414 (2.2)**
Other relationship problem (nonintimate)	296 (2.1)	107 (2.4)	**403 (2.1)**
Suicide of family member or friend during past 5 years	253 (1.8)	126 (2.8)	**379 (2.0)**
Victim of interpersonal violence during past month	35 (<1.0)	49 (1.1)	**84 (<1.0)**
**Life stressor**			
Crisis during previous or upcoming 2 weeks	4,386 (30.6)	1,139 (25.7)	**5,525 (29.4)**
Physical health problem	3,211 (22.4)	968 (21.9)	**4,179 (22.3)**
Argument or conflict	2,269 (15.8)	645 (14.6)	**2,914 (15.5)**
Job problem	1,564 (10.9)	295 (6.7)	**1,859 (9.9)**
Financial problem	1,315 (9.2)	351 (7.9)	**1,666 (8.9)**
Recent criminal legal problem	1,413 (9.9)	175 (4.0)	**1,588 (8.5)**
Noncriminal legal problem	569 (4.0)	179 (4.0)	**748 (4.0)**
Eviction or loss of home	560 (3.9)	162 (3.7)	**722 (3.8)**
School problem	183 (1.3)	60 (1.4)	**243 (1.3)**
History of child abuse/neglect	117 (<1.0)	112 (2.5)	**229 (1.2)**
Physical fight (two people, not a brawl)	121 (<1.0)	22 (<1.0)	**143 (<1.0)**
Traumatic anniversary	72 (<1.0)	43 (<1.0)	**115 (<1.0)**
Exposure to disaster	18 (<1.0)	4 (<1.0)	**22 (<1.0)**
Caretaker abuse/neglect led to suicide	3 (<1.0)	10 (<1.0)	**13 (<1.0)**
**Crime and criminal activity**			
Precipitated by another crime	531 (3.7)	41 (<1.0)	**572 (3.0)**
Crime in progress**	152 (28.6)	8 (19.5)	**160 (28.0)**
Terrorist attack	0 (0.0)	0 (0.0)	**0 (0.0)**
**Suicide event**			
Left a suicide note	4,685 (32.7)	1,783 (40.3)	**6,468 (34.5)**
History of suicidal thoughts or plans	4,408 (30.7)	1,582 (35.7)	**5,990 (31.9)**
History of suicide attempt(s)	2,304 (16.1)	1,428 (32.2)	**3,732 (19.9)**
**Suicide disclosure**			
Disclosed suicide intent	3,398 (23.7)	1,007 (22.7)	**4,405 (23.5)**
Disclosed intent to whom^††^			
Previous or current intimate partner	1,323 (38.9)	327 (32.5)	**1,650 (37.5)**
Other family member	968 (28.5)	333 (33.1)	**1,301 (29.5)**
Friend/colleague	399 (11.7)	127 (12.6)	**526 (11.9)**
Health care worker	141 (4.1)	70 (7.0)	**211 (4.8)**
Neighbor	50 (1.5)	12 (1.2)	**62 (1.4)**
Other person	287 (8.4)	70 (7.0)	**357 (8.1)**
Unknown	230 (6.8)	68 (6.8)	**298 (6.8)**
**Total^§§^**	**14,335 (91.3)**	**4,429 (93.4)**	**18,764 (91.8)**

**Abbreviations:** ADD/ADHD = attention deficit disorder/attention deficit hyperactivity disorder; OCD = obsessive-compulsive disorder; PTSD = posttraumatic stress disorder.

* Includes suicides with one or more precipitating circumstances. More than one circumstance could have been present per decedent.

^†^ Denominator is suicides with one or more precipitating circumstances. Therefore, the sums of percentages in columns exceed 100% because more than one circumstance could have been present per decedent.

^§^ Alaska, Arizona, Colorado, Connecticut, Georgia, Hawaii, Kansas, Kentucky, Maine, Maryland, Massachusetts, Michigan, Minnesota, New Hampshire, New Jersey, New Mexico, New York, North Carolina, Ohio, Oklahoma, Oregon, Rhode Island, South Carolina, Utah, Vermont, Virginia, and Wisconsin.

^¶^ Includes decedents with one or more current diagnosed mental health problems. Therefore, sums of percentages for the diagnosed conditions exceed 100%. Denominator is the number of decedents with one or more current diagnosed mental health problems.

** Denominator is decedents involved in an incident that was precipitated by another crime.

^††^ Denominator is decedents who disclosed intent.

^§§^ N = 20,446 (15,702 males and 4,744 females). Circumstances were unknown for 1,682 decedents (1,367 males and 315 females).

Among other circumstances related to suicide, 34.5% of decedents left a suicide note, 31.9% had a history of suicidal thoughts or plans, 19.9% had a history of previous suicide attempts, and 23.5% had disclosed suicidal intent to another person (Table 3). Of those who disclosed intent, the majority of disclosures were to a previous or current intimate partner (37.4%) or to some other family member (29.6%). Alcohol or other substance abuse problems were indicated for 17.4% and 16.4% of suicide decedents, respectively. A crisis during the preceding or upcoming 2 weeks (29.4%) and intimate partner problems (27.2%) were other common circumstances. Physical health problems (22.3%), an argument or conflict (15.5%), job or financial problems (9.9% and 8.9%, respectively), family relationship problems (8.9%), and recent criminal legal problems (8.5%) also were reported to have preceded suicides.

When examining known circumstances by sex, similar percentages of male and female decedents were reported to have a depressed mood at the time of death (Table 3). A greater percentage of female decedents was reported to have a current diagnosed mental health problem (66.2%) than male decedents (45.1%), and a greater percentage of female (41.1%) than male (23.2%) decedents was known to be receiving mental health treatment at the time of death. Suicide events, including leaving a suicide note, history of suicidal thoughts and plans, and history of suicide attempts, were indicated more frequently in female than male suicide cases.

### Homicides

#### Sex, Race/Ethnicity, and Age Group

The 27 NVDRS states included in this report collected data concerning 6,953 homicide incidents, which included 7,374 deaths in 2015 (Table 4). Overall, the crude homicide rate was 4.9 per 100,000 population. In more than half (52.3%) of homicides, the relationship of the victim to the suspect was not known; when the relationship was known, the suspect most often was an acquaintance/friend (28.1%), a spouse/intimate partner (21.5%), other person known to the victim (14.4%), or a stranger (12.5%).

**TABLE 4:** Number, percentage,* and rate^†^ of homicides, by decedent’s sex, age group, race/ethnicity, method used, location in which injury occurred, and victim-suspect relationship — National Violent Death Reporting System, 27 states,^§^ 2015

Characteristic	Male		Female		Total	
	No. (%)	Rate	No. (%)	Rate	No. (%)	Rate
**Age group (yrs)**						
<1	80 (1.4)	8.6	61 (3.9)	6.8	**141 (1.9)**	**7.7**
1–4	112 (1.9)	3.0	77 (5.0)	2.2	**189 (2.6)**	**2.6**
5–9	31 (<1.0)	0.6	29 (1.9)	0.6	**60 (<1.0)**	**0.6**
10–14	39 (<1.0)	0.8	23 (1.5)	0.5	**62 (<1.0)**	**0.6**
15–19	472 (8.1)	9.3	91 (5.9)	1.9	**563 (7.6)**	**5.7**
20–24	1,124 (19.3)	20.6	163 (10.6)	3.1	**1,287 (17.5)**	**12.1**
25–29	980 (16.8)	18.7	164 (10.6)	3.2	**1,144 (15.5)**	**11.1**
30–34	743 (12.7)	14.8	162 (10.5)	3.2	**905 (12.3)**	**9.0**
35–44	962 (16.5)	10.3	252 (16.3)	2.6	**1,214 (16.5)**	**6.4**
45–54	622 (10.7)	6.2	191 (12.4)	1.8	**813 (11.0)**	**4.0**
55–64	404 (6.9)	4.3	157 (10.2)	1.5	**561 (7.6)**	**2.9**
65–74	176 (3.0)	2.9	84 (5.4)	1.2	**260 (3.5)**	**2.0**
75–84	58 (<1.0)	2.1	64 (4.1)	1.7	**122 (1.7)**	**1.9**
≥85	24 (<1.0)	2.4	27 (1.7)	1.4	**51 (<1.0)**	**1.7**
Unknown	2 (<1.0)	—^¶^	0 (0.0)	—^¶^	**2 (<1.0)**	**—^¶^**
**Race/Ethnicity**						
White, non-Hispanic	1,416 (24.3)	2.8	768 (49.7)	1.5	**2,184 (29.6)**	**2.2**
Black, non-Hispanic	3,489 (59.9)	35.0	511 (33.1)	4.7	**4,000 (54.2)**	**19.1**
American Indian/Alaska Native, non-Hispanic	122 (2.1)	14.6	45 (2.9)	5.2	**167 (2.3)**	**9.8**
Asian/Pacific Islander	88 (1.5)	2.2	38 (2.5)	0.9	**126 (1.7)**	**1.5**
Hispanic**	685 (11.8)	7.4	173 (11.2)	1.9	**858 (11.6)**	**4.7**
Other	26 (<1.0)	—^¶^	8 (<1.0)	—^¶^	**34 (<1.0)**	**—^¶^**
Unknown	3 (<1.0)	—^¶^	2 (<1.0)	—^¶^	**5 (<1.0)**	**—^¶^**
**Method**						
Firearm	4,381 (75.2)	5.9	803 (52.0)	1.0	**5,184 (70.3)**	**3.4**
Sharp instrument	650 (11.2)	0.9	237 (15.3)	0.3	**887 (12.0)**	**0.6**
Personal weapons (e.g., hands, feet, fists)	248 (4.3)	0.3	104 (6.7)	0.1	**352 (4.8)**	**0.2**
Blunt instrument	232 (4.0)	0.3	118 (7.6)	0.2	**350 (4.7)**	**0.2**
Hanging/strangulation/suffocation	90 (1.5)	0.1	120 (7.8)	0.2	**210 (2.8)**	**0.1**
Motor vehicles (e.g., buses, motorcycles, other transport vehicles)	38 (<1.0)	0.1	25 (1.6)	0.0	**63 (<1.0)**	**0.0**
Fire/burns	22 (<1.0)	0.0	21 (1.4)	0.0	**43 (<1.0)**	**0.0**
Poisoning	27 (<1.0)	0.0	16 (1.0)	—^¶^	**43 (<1.0)**	**0.0**
Intentional neglect	13 (<1.0)	—^¶^	15 (<1.0)	—^¶^	**28 (<1.0)**	**0.0**
Drowning	15 (<1.0)	—^¶^	10 (<1.0)	—^¶^	**25 (<1.0)**	**0.0**
Fall	17 (<1.0)	—^¶^	8 (<1.0)	—^¶^	**25 (<1.0)**	**0.0**
Other (single method)	22 (<1.0)	—^¶^	11 (<1.0)	—^¶^	**33 (<1.0)**	**—^¶^**
Unknown	74 (1.3)	—^¶^	57 (3.7)	—^¶^	**131 (1.8)**	**—^¶^**
**Location**						
House/apartment	2,525 (43.3)	3.4	1,086 (70.3)	1.4	**3,611 (49.0)**	**2.4**
Street/highway	1,532 (26.3)	2.1	113 (7.3)	0.1	**1,645 (22.3)**	**1.1**
Motor vehicle	436 (7.5)	0.6	77 (5.0)	0.1	**513 (7.0)**	**0.3**
Parking lot/public garage/public transport	304 (5.2)	0.4	31 (2.0)	0.0	**335 (4.5)**	**0.2**
Commercial/retail area	212 (3.6)	0.3	18 (1.2)	—^¶^	**230 (3.1)**	**0.2**
Natural area	103 (1.8)	0.1	33 (2.1)	0.0	**136 (1.8)**	**0.1**
Bar/nightclub	119 (2.0)	0.2	4 (<1.0)	—^¶^	**123 (1.7)**	**0.1**
Other location^††^	360 (6.2)	—^¶^	104 (6.7)	—^¶^	**464 (6.3)**	**—^¶^**
Unknown	238 (4.1)	—^¶^	79 (5.1)	—^¶^	**317 (4.3)**	**—^¶^**
**Relationship^§§^**						
Acquaintance/friend	853 (35.2)	1.2	133 (12.1)	0.2	**986 (28.1)**	**0.7**
Spouse/intimate partner (current or former)	192 (7.9)	0.3	563 (51.4)	0.7	**755 (21.5)**	**0.5**
Other person known to victim	435 (18.0)	—^¶^	72 (6.6)	—^¶^	**507 (14.4)**	**—^¶^**
Stranger	390 (16.1)	0.5	50 (4.6)	0.1	**440 (12.5)**	**0.3**
Other relative	172 (7.1)	—^¶^	61 (5.6)	—^¶^	**233 (6.6)**	**—^¶^**
Child	140 (5.8)	0.2	90 (8.2)	0.1	**230 (6.5)**	**0.2**
Parent	100 (4.1)	0.1	84 (7.7)	0.1	**184 (5.2)**	**0.1**
Other intimate partner involvement^¶¶^	63 (2.6)	—^¶^	40 (3.7)	—^¶^	**103 (2.9)**	**—^¶^**
Rival gang member	63 (2.6)	0.1	2 (<1.0)	—^¶^	**65 (1.8)**	**0.0**
Victim was law enforcement officer injured in the line of duty	10 (<1.0)	—^¶^	0 (0.0)	—^¶^	**10 (<1.0)**	**—^¶^**
Victim was injured by a law enforcement officer	2 (<1.0)	—^¶^	0 (0.0)	—^¶^	**2 (<1.0)**	**—^¶^**
**Total**	**5,829 (100)**	**7.9**	**1,545 (100)**	**2.0**	**7,374 (100)**	**4.9**

* Percentages might not total 100% due to rounding.

^†^ Per 100,000 population.

^§^ Alaska, Arizona, Colorado, Connecticut, Georgia, Hawaii, Kansas, Kentucky, Maine, Maryland, Massachusetts, Michigan, Minnesota, New Hampshire, New Jersey, New Mexico, New York, North Carolina, Ohio, Oklahoma, Oregon, Rhode Island, South Carolina, Utah, Vermont, Virginia, and Wisconsin.

^¶^ Rates not reported when number of decedents is <20 or when characteristic response is other or unknown.

** Includes persons of any race.

^††^ Includes (in descending order) hotel/motel, park/playground/sports or athletic area, jail/prison, abandoned house/building/warehouse, hospital or medical facility, preschool/school/college/school bus, supervised residential facility, office building, industrial or construction area, farm, railroad tracks, and other unspecified location.

^§§^ Percentage is based on the number of homicide decedents with a known victim-suspect relationship (n = 3,515 [2,420 males and 1,095 females]). Victim-suspect relationship was unknown for 3,859 decedents.

^¶¶^ Death was related to intimate partner violence but not between the intimate partners (e.g., a child killed by mother’s boyfriend).

The homicide rate for males was nearly four times the rate for females (7.9 and 2.0 per 100,000 population, respectively) and approximately five times the rate for females among those aged 20–29 years (Table 4). When the relationship of the victim to suspect was known, among male decedents the suspect was most frequently an acquaintance/friend (35.2%) and among female decedents the suspect was most frequently a current or former spouse/intimate partner (51.4%). Non-Hispanic blacks accounted for more than half (54.2%) of homicides and had the highest rate (19.1 per 100,000 population), followed by non-Hispanic American Indian/Alaska Natives (9.8 per 100,000 population) and Hispanics (4.7 per 100,000 population). Non-Hispanic black males had the highest rate of homicide deaths of any racial/ethnic group (35.0 per 100,000 population). This rate was 12.5 times the homicide rate for non-Hispanic white males (2.8 per 100,000), approximately two times the homicide rate for American Indian/Alaska Native males (14.6 per 100,000 population), and four times the homicide rate for Hispanic males (7.4 per 100,000 population). Age-specific homicide rates were highest among persons aged 20–24 years (12.1 per 100,000 population), followed by persons aged 25–29 years (11.1 per 100,000 population). The rate for infants aged <1 year was more than 2.5 times the rate for children aged 1–4 years (7.7 and 2.6 per 100,000 population, respectively). Rates were lowest among persons aged 5–14 years and ≥65 years. Among male homicide decedents, the majority (65.3%) were aged 20–44 years; the rate was highest among those aged 20–24 years (20.6 per 100,000 population). Among female homicide decedents, nearly half (48.0%) were aged 20–44 years; the homicide rate was highest among infants aged <1 year (6.8 per 100,000 population). Among male infants aged <1 year, the homicide rate was 8.6 per 100,000 population.

#### Method and Location of Injury

Firearms were used in 70.3% of homicides, followed by sharp instruments (12.0%), personal weapons (e.g., hands, feet, or fists) (4.8%), blunt instruments (4.7%), and hanging/strangulation/suffocation (2.8%) (Table 4). No other method was used in more than 1% of homicides. Firearms were the most common method used in homicides of males and females (75.2% and 52.0%, respectively). Sharp instruments were more common among females than males (15.3% and 11.2%, respectively), as were personal weapons (6.7% and 4.3%, respectively), blunt instruments (7.6% and 4.0%, respectively), and hanging/strangulation/suffocation (7.8% and 1.5%, respectively). A house/apartment was the most common location of homicide (49.0%), followed by a street/highway (22.3%), a motor vehicle (7.0%), and a parking lot/public garage/public transport (4.5%). Homicides occurred with greater frequency at a house/apartment among females than males (70.3% and 43.3%, respectively), whereas homicides occurred on a street/highway approximately 3.5 times more frequently among males than females (26.3% and 7.3%, respectively).

#### Precipitating Circumstances

Precipitating circumstances were identified for 80.4% of homicides (Table 5). Approximately one in three homicides with known circumstances was precipitated by another crime (34.7%); in 54.4% of those cases the crime was in progress at the time of the incident. The type of crime most frequently precipitating the homicide was assault (46.2%), followed by robbery (30.7%), drug trade** (13.5%), burglary (12.1%), motor vehicle theft (2.6%), rape/sexual assault (2.0%), and arson (1.8%). An argument or conflict (34.9%), a physical fight between two people (14.1%), drug involvement (11.5%), or a substance abuse problem other than alcohol abuse (11.0%) were other common precipitating circumstances. In 16.8% of homicides with known circumstances, intimate partner violence (IPV) was identified as a contributing factor.

**TABLE 5:** Number* and percentage^†^ of homicides, by precipitating circumstances and decedent’s sex — National Violent Death Reporting System, 27 states,^§^ 2015

Precipitating circumstance	Male	Female	Total
	No. (%)	No. (%)	No. (%)
**Mental health/Substance abuse**			
Substance abuse problem (excludes alcohol)	518 (11.3)	134 (9.9)	**652 (11.0)**
Current diagnosed mental health problem	173 (3.8)	86 (6.4)	**259 (4.4)**
Alcohol problem	194 (4.2)	49 (3.6)	**243 (4.1)**
History of ever being treated for a mental health problem	116 (2.5)	54 (4.0)	**170 (2.9)**
Current mental health treatment	76 (1.7)	38 (2.8)	**114 (1.9)**
Current depressed mood	15 (<1.0)	18 (1.3)	**33 (<1.0)**
Other addiction (e.g., gambling, sex)	7 (<1.0)	4 (<1.0)	**11 (<1.0)**
**Interpersonal**			
Intimate partner violence–related	384 (8.4)	613 (45.4)	**997 (16.8)**
Family relationship problem	206 (4.5)	118 (8.7)	**324 (5.5)**
Other relationship problem (nonintimate)	243 (5.3)	39 (2.9)	**282 (4.8)**
Jealousy (lovers’ triangle)	112 (2.4)	59 (4.4)	**171 (2.9)**
Victim of interpersonal violence during past month	44 (<1.0)	65 (4.8)	**109 (1.8)**
Perpetrator of interpersonal violence during past month	77 (1.7)	8 (<1.0)	**85 (1.4)**
**Life stressor**			
Argument or conflict	1,674 (36.5)	394 (29.2)	**2,068 (34.9)**
Physical fight (two people, not a brawl)	738 (16.1)	100 (7.4)	**838 (14.1)**
Crisis during previous or upcoming 2 weeks	309 (6.7)	167 (12.4)	**476 (8.0)**
History of child abuse/neglect	42 (<1.0)	33 (2.4)	**75 (1.3)**
**Crime and criminal activity**			
Precipitated by another crime	1,685 (36.8)	372 (27.6)	**2,057 (34.7)**
Crime in progress^¶^	929 (55.1)	189 (50.8)	**1,118 (54.4)**
Drug involvement	601 (13.1)	82 (6.1)	**683 (11.5)**
Gang-related	379 (8.3)	24 (1.8)	**403 (6.8)**
Terrorist attack	2 (<1.0)	1 (<1.0)	**3 (<1.0)**
**Homicide event**			
Caretaker abuse/neglect led to death	193 (4.2)	149 (11.0)	**342 (5.8)**
Victim used a weapon	295 (6.4)	12 (<1.0)	**307 (5.2)**
Drive-by shooting	229 (5.0)	28 (2.1)	**257 (4.3)**
Mentally ill suspect	102 (2.2)	98 (7.3)	**200 (3.4)**
Walk-by assault	176 (3.8)	15 (1.1)	**191 (3.2)**
Justifiable self-defense	156 (3.4)	3 (<1.0)	**159 (2.7)**
Random violence	117 (2.6)	23 (1.7)	**140 (2.4)**
Victim was a bystander	88 (1.9)	47 (3.5)	**135 (2.3)**
Brawl	106 (2.3)	14 (1.0)	**120 (2.0)**
Victim was an intervener assisting a crime victim	51 (1.1)	8 (<1.0)	**59 (<1.0)**
Prostitution	11 (<1.0)	18 (1.3)	**29 (<1.0)**
Stalking	4 (<1.0)	15 (1.1)	**19 (<1.0)**
Victim was a police officer on duty	14 (<1.0)	0 (0.0)	**14 (<1.0)**
Hate crime	8 (<1.0)	6 (<1.0)	**14 (<1.0)**
Mercy killing	3 (<1.0)	9 (<1.0)	**12 (<1.0)**
Total**	**4,582 (78.6)**	**1,349 (87.3)**	**5,931 (80.4)**

* Includes homicides with one or more precipitating circumstances. Totals do not equal the sums of the columns because more than one circumstance could have been present per decedent.

^†^ Denominator is homicides with one or more precipitating circumstances. The sum of percentages in columns exceeds 100% because more than one circumstance could have been present per decedent.

^§^ Alaska, Arizona, Colorado, Connecticut, Georgia, Hawaii, Kansas, Kentucky, Maine, Maryland, Massachusetts, Michigan, Minnesota, New Hampshire, New Jersey, New Mexico, New York, North Carolina, Ohio, Oklahoma, Oregon, Rhode Island, South Carolina, Utah, Vermont, Virginia, and Wisconsin.

^¶^ Denominator is decedents involved in an incident that was precipitated by another crime.

** N = 7,374 (5,829 males and 1,545 females). Circumstances were unknown for 1,443 decedents (1,247 males and 196 females).

Among all reported homicide circumstances, IPV accounted for the largest percentage difference by sex. IPV was a known circumstance for approximately 45.4% of homicides among females but only 8.4% of homicides among males (Table 5). An argument or conflict was a factor in 36.5% of homicides among males but only 29.2% of homicides among females. Physical fights precipitated 16.1% of homicides among males but only 7.4% among females. Similarly, drug involvement more commonly precipitated homicide among males, contributing to 13.1% of homicides among males and 6.1% among females. A recent crisis (during the previous or upcoming 2 weeks) was implicated in 12.6% of homicides among females but only 6.7% among males. Gang-related homicides were more common among males (8.3%) than females (1.8%). Male decedents used a weapon during the incident in 6.4% and female decedents in <1.0% of homicides with known circumstances.

### Legal Intervention Deaths

#### Sex, Race/Ethnicity, and Age Group

The 27 NVDRS states included in this report collected data concerning 416 legal intervention death incidents, which included 417 deaths in 2015 (Table 6). Almost all legal intervention deaths occurring in 2015 were among males (96.6%). The highest rate was among males aged 30–34 years (1.2 per 100,000 population), followed by those aged 20–24 years (1.0 per 100,000 population), 25–29 years (1.0 per 100,000 population), and 35–44 years (0.9 per 100,000 population). Non-Hispanic white males accounted for the highest percentage of legal intervention deaths (55.3%), whereas non-Hispanic black males had the highest rate (1.0 per 100,000 population), 2.5 times the rate for non-Hispanic white males (0.4 per 100,000). Of the 403 male decedents, 13.2% were Hispanic; their crude legal intervention death rate was 0.6 per 100,000 population.

**TABLE 6:** Number, percentage,* and rate^†^ of legal intervention^§^ deaths, by decedent’s sex, age group, race/ethnicity, method used, and location in which injury occurred — National Violent Death Reporting System, 27 states,^¶^ 2015

Characteristic	Male		Female		Total	
	No. (%)	Rate	No. (%)	Rate	No. (%)	Rate
**Age group (yrs)**						
<1	0 (0.0)	—**	0 (0.0)	—**	**0 (0.0)**	—**
1–4	0 (0.0)	—**	0 (0.0)	—**	**0 (0.0)**	—**
5–9	0 (0.0)	—**	0 (0.0)	—**	**0 (0.0)**	—**
10–14	0 (0.0)	—**	0 (0.0)	—**	**0 (0.0)**	—**
15–19	24 (6.0)	0.5	1 (7.1)	—**	**25 (6.0)**	**0.3**
20–24	55 (13.6)	1.0	2 (14.3)	—**	**57 (13.7)**	**0.5**
25–29	52 (12.9)	1.0	3 (21.4)	—**	**55 (13.2)**	**0.5**
30–34	60 (14.9)	1.2	0 (0.0)	—**	**60 (14.4)**	**0.6**
35–44	84 (20.8)	0.9	4 (28.6)	—**	**88 (21.1)**	**0.5**
45–54	71 (17.6)	0.7	3 (21.4)	—**	**74 (17.7)**	**0.4**
55–64	39 (9.7)	0.4	1 (7.1)	—**	**40 (9.6)**	**0.2**
65–74	13 (3.2)	—**	0 (0.0)	—**	**13 (3.1)**	—**
75–84	4 (<1.0)	—**	0 (0.0)	—**	**4 (<1.0)**	—**
≥85	1 (<1.0)	—**	0 (0.0)	—**	**1 (<1.0)**	—**
Unknown	0 (0.0)	—**	0 (0.0)	—**	**0 (0.0)**	—**
**Race/Ethnicity**						
White, non-Hispanic	223 (55.3)	0.4	8 (57.1)	—**	**231 (55.4)**	**0.2**
Black, non-Hispanic	100 (24.8)	1.0	3 (21.4)	—**	**103 (24.7)**	**0.5**
American Indian/Alaska Native, non-Hispanic	17 (4.2)	—**	0 (0.0)	—**	**17 (4.1)**	—**
Asian/Pacific Islander	6 (1.5)	—**	0 (0.0)	—**	**6 (1.4)**	—**
Hispanic^††^	53 (13.2)	0.6	3 (21.4)	—**	**56 (13.4)**	**0.3**
Other	3 (<1.0)	—**	0 (0.0)	—**	**3 (<1.0)**	—**
Unknown	1 (<1.0)	—**	0 (0.0)	—**	**1 (<1.0)**	—**
**Method**						
Firearm	377 (93.5)	0.5	9 (64.3)	—**	**386 (92.6)**	**0.3**
Motor vehicles (e.g., buses, motorcycles, other transport vehicles)	6 (1.5)	—**	3 (21.4)	—**	**9 (2.2)**	—**
Personal weapons (e.g., hands, feet, fists)	3 (<1.0)	—**	0 (0.0)	—**	**3 (<1.0)**	—**
Blunt instrument	1 (<1.0)	—**	0 (0.0)	—**	**1 (<1.0)**	—**
Drowning	1 (<1.0)	—**	0 (0.0)	—**	**1 (<1.0)**	—**
Hanging/strangulation/suffocation	1 (<1.0)	—**	0 (0.0)	—**	**1 (<1.0)**	—**
Sharp instrument	0 (0.0)	—**	1 (7.1)	—**	**1 (<1.0)**	—**
Fall	0 (0.0)	—**	0 (0.0)	—**	**0 (0.0)**	—**
Fire/burns	0 (0.0)	—**	0 (0.0)	—**	**0 (0.0)**	—**
Intentional neglect	0 (0.0)	—**	0 (0.0)	—**	**0 (0.0)**	—**
Poisoning	0 (0.0)	—**	0 (0.0)	—**	**0 (0.0)**	—**
Other (single method)	13 (3.2)	—**	0 (0.0)	—**	**13 (3.1)**	—**
Unknown	1 (<1.0)	—**	1 (7.1)	—**	**2 (<1.0)**	—**
**Location**						
House/apartment	170 (42.2)	0.2	6 (42.9)	—**	**176 (42.2)**	**0.1**
Street/highway	109 (27.0)	0.1	1 (7.1)	—**	**110 (26.4)**	**0.1**
Motor vehicle	32 (7.9)	0.0	4 (28.6)	—**	**36 (8.6)**	**0.0**
Parking lot/public garage/public transport	28 (6.9)	0.0	0 (0.0)	—**	**28 (6.7)**	**0.0**
Natural area	18 (4.5)	—**	1 (7.1)	—**	**19 (4.6)**	—**
Other location^§§^	44 (10.9)	—**	2 (14.3)	—**	**46 (11.0)**	—**
Unknown	2 (<1.0)	—**	0 (0.0)	—**	**2 (<1.0)**	—**
**Total**	**403 (100)**	**0.5**	**14 (100)**	—**	**417 (100)**	**0.3**

* Percentages might not total 100% due to rounding.

^†^ Per 100,000 population.

^§^ The term “legal intervention” does not denote the lawfulness or legality of the circumstances surrounding the death.

^¶^ Alaska, Arizona, Colorado, Connecticut, Georgia, Hawaii, Kansas, Kentucky, Maine, Maryland, Massachusetts, Michigan, Minnesota, New Hampshire, New Jersey, New Mexico, New York, North Carolina, Ohio, Oklahoma, Oregon, Rhode Island, South Carolina, Utah, Vermont, Virginia, and Wisconsin.

** Rates not reported when number of decedents is <20 or when characteristic response is other or unknown.

^††^ Includes persons of any race.

^§§^ Includes commercial/retail area, hotel/motel, jail/prison, office building, bar/nightclub, preschool/school/college/school bus, farm, supervised residential facility, and other unspecified location.

#### Method and Location of Injury

Firearms were used in the majority (92.6%) of legal intervention deaths (Table 6). Legal intervention deaths occurred most frequently in a house/apartment (42.2%), followed by a street/highway (26.4%) and a motor vehicle (8.6%).

#### Precipitating Circumstances

Precipitating circumstances were identified for 99.0% of legal intervention deaths (Table 7). Approximately 86.0% were precipitated by another crime; in 61.4% of these, the crime was in progress at the time of the incident. Assault/homicide (54.9%), followed by robbery (6.5%), motor vehicle theft (6.2%), burglary (4.5%), drug trade (2.5%), and arson (1.1%) were the types of crimes most frequently precipitating the death. The decedent reportedly used a weapon in 70.7% of cases. In 21.8% of legal intervention deaths with known circumstances, substance abuse problems (other than alcohol) was reported as a contributing factor. In 18.4% of legal intervention deaths, the decedent had a current diagnosed mental health problem. An argument or conflict (16.2%), being a perpetrator of interpersonal violence during the past month (6.1%), family relationship problems (6.5%), and drug involvement (5.8%) were other notable precipitating circumstances. Among legal intervention deaths with known circumstances, IPV was identified as a contributing factor in 10.7% and a recent crisis (during the previous or upcoming 2 weeks) was cited in 12.8%.

**TABLE 7:** Number* and percentage^†^ of legal intervention^§^ deaths, by precipitating circumstances and decedent’s sex — National Violent Death Reporting System, 27 states,^¶^ 2015

Precipitating circumstance	Male	Female	Total
	No. (%)	No. (%)	No. (%)
**Mental health/Substance abuse**			
Substance abuse problem (excludes alcohol)	87 (21.8)	3 (23.1)	**90 (21.8)**
Current diagnosed mental health problem	72 (18.0)	4 (30.8)	**76 (18.4)**
History of ever being treated for a mental health problem	61 (15.3)	4 (30.8)	**65 (15.7)**
Current mental health treatment	35 (8.8)	4 (30.8)	**39 (9.4)**
Alcohol problem	38 (9.5)	1 (7.7)	**39 (9.4)**
Current depressed mood	25 (6.3)	1 (7.7)	**26 (6.3)**
Other addiction (e.g., gambling, sex)	4 (1.0)	0 (0.0)	**4 (<1.0)**
**Interpersonal**			
Intimate partner violence-related	42 (10.5)	2 (15.4)	**44 (10.7)**
Family relationship problem	27 (6.8)	0 (0.0)	**27 (6.5)**
Perpetrator of interpersonal violence during past month	25 (6.3)	0 (0.0)	**25 (6.1)**
Other relationship problem (nonintimate)	11 (2.8)	1 (7.7)	**12 (2.9)**
Jealousy (lovers’ triangle)	4 (1.0)	0 (0.0)	**4 (<1.0)**
Victim of interpersonal violence during past month	0 (0.0)	0 (0.0)	**0 (0.0)**
**Life stressor**			
Argument or conflict	65 (16.3)	2 (15.4)	**67 (16.2)**
Crisis during previous or upcoming 2 weeks	53 (13.3)	0 (0.0)	**53 (12.8)**
Physical fight (two people, not a brawl)	27 (6.8)	0 (0.0)	**27 (6.5)**
History of child abuse/neglect	0 (0.0)	0 (0.0)	**0 (0.0)**
**Crime and criminal activity**			
Precipitated by another crime	344 (86.0)	11 (84.6)	**355 (86.0)**
Crime in progress**	210 (61.0)	8 (72.7)	**218 (61.4)**
Drug involvement	23 (5.8)	1 (7.7)	**24 (5.8)**
Gang-related	5 (1.3)	0 (0.0)	**5 (1.2)**
Terrorist attack	0 (0.0)	0 (0.0)	**0 (0.0)**
**Legal intervention event**			
Victim used a weapon	286 (71.5)	6 (46.2)	**292 (70.7)**
Brawl	7 (1.8)	0 (0.0)	**7 (1.7)**
Victim was a bystander	1 (<1.0)	1 (7.7)	**2 (<1.0)**
Stalking	2 (<1.0)	0 (0.0)	**2 (<1.0)**
Victim was an intervener assisting a crime victim	1 (<1.0)	0 (0.0)	**1 (<1.0)**
Victim was a police officer on duty	0 (0.0)	0 (0.0)	**0 (0.0)**
Mentally ill suspect	0 (0.0)	0 (0.0)	**0 (0.0)**
Random violence	0 (0.0)	0 (0.0)	**0 (0.0)**
Prostitution	0 (0.0)	0 (0.0)	**0 (0.0)**
**Total^††^**	**400 (99.3)**	**13 (92.9)**	**413 (99.0)**

* Includes deaths with one or more precipitating circumstances. Totals do not equal the sums of the columns because more than one circumstance could have been present per decedent.

^†^ Denominator is deaths with one or more precipitating circumstances. The sum of percentages in columns exceed 100% because more than one circumstance could have been present per decedent.

^§^ The term “legal intervention” does not denote the lawfulness or legality of the circumstances surrounding the death.

^¶^ Alaska, Arizona, Colorado, Connecticut, Georgia, Hawaii, Kansas, Kentucky, Maine, Maryland, Massachusetts, Michigan, Minnesota, New Hampshire, New Jersey, New Mexico, New York, North Carolina, Ohio, Oklahoma, Oregon, Rhode Island, South Carolina, Utah, Vermont, Virginia, and Wisconsin.

** Denominator is decedents involved in an incident that was precipitated by another crime.

^††^ N = 417 (403 males and 14 females). Circumstances were unknown for four decedents (three males and one female).

### Unintentional Firearm Deaths

#### Sex, Race/Ethnicity, and Age Group

The 27 NVDRS states included in this report collected data concerning 204 incidents involving 204 unintentional firearm injury deaths in 2015 (Table 8). Approximately half (50.5%) of these deaths were self-inflicted and 85 (41.7%) were known to be inflicted by another person; for the remaining 16 (7.8%), who inflicted the injury was not known. Males accounted for 81.4% of decedents. The majority were non-Hispanic whites (64.2%), followed by non-Hispanic blacks (22.5%). Persons aged ≤24 years accounted for more than half (51.5%) of all unintentional firearm deaths.

**TABLE 8:** Number and percentage* of unintentional firearm deaths, by decedent’s sex, race/ethnicity, age group, location in which injury occurred, and type of firearm — National Violent Death Reporting System, 27 states,^†^ 2015

Characteristic	No. (%)
**Sex**	
Male	166 (81.4)
Female	38 (18.6)
**Race/Ethnicity**	
White, non-Hispanic	131 (64.2)
Black, non-Hispanic	46 (22.5)
American Indian/Alaska Native, non-Hispanic	10 (4.9)
Asian/Pacific Islander	2 (<1.0)
Hispanic^§^	15 (7.4)
**Age group (yrs)**	
1–4	19 (9.3)
5–9	5 (2.5)
10–14	14 (6.9)
15–19	38 (18.6)
20–24	29 (14.2)
25–29	24 (11.8)
30–34	10 (4.9)
35–44	19 (9.3)
45–54	14 (6.9)
55–64	17 (8.3)
65–74	7 (3.4)
75–84	7 (3.4)
≥85	1 (<1.0)
**Location**	
House/apartment	164 (80.4)
Natural area	20 (9.8)
Motor vehicle	6 (2.9)
Farm	4 (2.0)
Street/highway	4 (2.0)
Commercial/retail area	2 (<1.0)
Other unspecified location^¶^	1 (<1.0)
Unknown	3 (1.5)
**Firearm type**	
Handgun	126 (61.8)
Rifle	35 (17.2)
Shotgun	20 (9.8)
Unknown	23 (11.3)
**Total**	**204 (100)**

* Percentages might not total 100% due to rounding.

^†^ Alaska, Arizona, Colorado, Connecticut, Georgia, Hawaii, Kansas, Kentucky, Maine, Maryland, Massachusetts, Michigan, Minnesota, New Hampshire, New Jersey, New Mexico, New York, North Carolina, Ohio, Oklahoma, Oregon, Rhode Island, South Carolina, Utah, Vermont, Virginia, and Wisconsin.

^§^ Includes persons of any race.

^¶^ Includes military training exercise, private land campsites, and private hunting land attached to homes.

#### Location of Injury and Firearm Type

Of all unintentional firearm deaths, 80.4% occurred in a house/apartment, followed by natural areas (9.8%) and a motor vehicle (2.9%) (Table 8). The majority of unintentional firearm injury deaths involved a handgun (61.8%), followed by a rifle (17.2%) and a shotgun (9.8%). In 11.3% of deaths, the firearm type was unknown.

#### Context of Injury and Associated Circumstances

The context of the injury or associated circumstances was known for 96.6% of unintentional firearm deaths (Table 9). Overall, the most common context of injury was playing with a gun (39.1%), followed by hunting (11.2%), cleaning the gun (9.6%), and showing the gun to others (8.1%). Unintentionally pulling the trigger (21.3%) was the most common associated circumstance, followed by mistakenly thinking the gun was unloaded (15.2%) and mistakenly thinking the magazine was disengaged (7.1%).

**TABLE 9:** Number and percentage* of unintentional firearm deaths, by context and circumstances of injury — National Violent Death Reporting System, 27 states,^†^ 2015

Characteristic	No. (%)
**Context of injury**	
Playing with gun	77 (39.1)
Hunting	22 (11.2)
Cleaning gun	19 (9.6)
Showing gun to others	16 (8.1)
Loading/unloading gun	12 (6.1)
Target shooting	7 (3.6)
Celebratory firing	1 (<1.0)
Other context of injury	44 (22.3)
**Circumstance of injury**	
Unintentionally pulled trigger	42 (21.3)
Thought gun was unloaded	30 (15.2)
Thought unloaded, magazine disengaged	14 (7.1)
Gun was dropped	13 (6.6)
Gun fired due to defect or malfunction	8 (4.1)
Gun was mistaken for a toy	6 (3.0)
Thought gun safety was engaged	3 (1.5)
Gun fired while holstering	3 (1.5)
Gun fired while handling safety lock	2 (1.0)
Bullet ricocheted	1 (<1.0)
Other mechanism of injury	24 (12.2)
**Total^§^**	**197 (96.6)**

* Percentages might exceed 100% because one or more circumstances could have been known per death. Therefore, number and percentage are reported when the number of deaths is <5 because no particular circumstance identifies a single death.

^†^ Alaska, Arizona, Colorado, Connecticut, Georgia, Hawaii, Kansas, Kentucky, Maine, Maryland, Massachusetts, Michigan, Minnesota, New Hampshire, New Jersey, New Mexico, New York, North Carolina, Ohio, Oklahoma, Oregon, Rhode Island, South Carolina, Utah, Vermont, Virginia, and Wisconsin.

^§^ N = 204. Circumstances were unknown for seven decedents.

### Deaths of Undetermined Intent

#### Sex, Race/Ethnicity, and Age Group

The 27 NVDRS states included in this report collected data concerning 2,956 incidents involving 2,974 deaths in 2015 for which a determination of intent could not be made. The overall crude rate of deaths of undetermined intent was 2.0 per 100,000 population. Rates were higher among males than among females (2.5 and 1.4 per 100,000 population, respectively). Non-Hispanic whites accounted for 70.6% of deaths, whereas non-Hispanic American Indian/Alaska Natives had the highest rate (3.6 per 100,000 population). Among males, non-Hispanic American Indian/Alaska Natives had the highest rate (4.9 per 100,000 population), followed by non-Hispanic blacks (3.7 per 100,000 population). Approximately 70.9% of persons for whom the manner of death was undetermined were aged 30–64 years. Rates were highest among adults aged 45–54 years (3.4 per 100,000 population), followed by adults aged 35–44 years (3.2 per 100,000 population) and 30–34 years (3.1 per 100,000 population).

#### Method and Location of Injury

Poisoning was the most common method of injury in deaths of undetermined intent (68.3%). No other method accounted for >4% overall. The majority of deaths of undetermined intent occurred in a house/apartment (69.3%), followed by a natural area (5.2%), street/highway (3.8%), and hotel/motel (3.2%).

#### Precipitating Circumstances

Precipitating circumstances were known in 84.4% of deaths of undetermined intent. Of those, substance abuse problems (other than alcohol) (63.6%) and alcohol problems (28.3%) were the most common. Current depressed mood was reported for 11.1% of decedents, and 26.0% were receiving mental health treatment at the time of their death. Of those decedents with a current diagnosed mental health problem (41.2%), depression/dysthymia (59.3%), anxiety disorder (19.8%), and bipolar disorder (22.4%) were the most common diagnoses. Among decedents, 10.2% had a history of suicide attempts, 11.6% had a history of suicidal thoughts or plans, 5.5% had disclosed intent to die by suicide, and 1.9% had left a suicide note. Physical health problems (15.2%) and a crisis during the preceding or upcoming 2 weeks (11.9%) were other circumstances identified in deaths of undetermined intent.

### Suicides Among Military Personnel

#### Sex, Race/Ethnicity, and Age Group

The 27 NVDRS states included in this report collected data concerning 3,429 suicides by current or former military personnel that occurred during 2015 (Table 10). Of these decedents, the majority were male (96.4%) and non-Hispanic white (89.6%). More than half of decedents (52.0%) were aged 45–74 years and 15% were aged 75–84 years.

**TABLE 10:** Number* and percentage^†^ of suicides among military personnel,^§^ by sex, age group, race/ethnicity, method used, and location in which injury occurred — National Violent Death Reporting System, 27 states,^¶^ 2015

Characteristic	No. (%)
**Sex**	
Male	3,305 (96.4)
Female	124 (3.6)
**Age group (yrs)**	
<20	15 (<1.0)
20–24	121 (3.5)
25–29	191 (5.6)
30–34	180 (5.2)
35–44	334 (9.7)
45–54	528 (15.4)
55–64	555 (16.2)
65–74	700 (20.4)
75–84	513 (15.0)
≥85	292 (8.5)
**Race/Ethnicity**	
White, non-Hispanic	3,073 (89.6)
Black, non-Hispanic	181 (5.3)
American Indian/Alaska Native, non-Hispanic	37 (1.1)
Asian/Pacific Islander	41 (1.2)
Hispanic**	91 (2.7)
Other	5 (<1.0)
Unknown	1 (<1.0)
**Method**	
Firearm	2,381 (69.4)
Hanging/strangulation/suffocation	569 (16.6)
Poisoning	315 (9.2)
Sharp instrument	61 (1.8)
Fall	36 (1.0)
Motor vehicles (e.g., buses, motorcycles, other transport vehicles)	26 (<1.0)
Drowning	20 (<1.0)
Fire/burns	9 (<1.0)
Blunt instrument	2 (<1.0)
Intentional neglect	0 (0.0)
Personal weapons (e.g., hands, feet, fists)	0 (0.0)
Other (single method)	3 (<1.0)
Unknown	7 (<1.0)
**Location**	
House/apartment	2,648 (77.2)
Motor vehicle	155 (4.5)
Natural area	153 (4.5)
Parking lot/public garage/public transport	80 (2.3)
Street/highway	80 (2.3)
Hotel/motel	63 (1.8)
Park/playground/sports or athletic area	51 (1.5)
Jail/prison	20 (<1.0)
Hospital or medical facility	19 (<1.0)
Commercial/retail area	16 (<1.0)
Supervised residential facility	11 (<1.0)
Farm	10 (<1.0)
Office building	9 (<1.0)
Railroad tracks	8 (<1.0)
Industrial or construction area	4 (<1.0)
Preschool/school/college/school bus	4 (<1.0)
Abandoned house/building/warehouse	1 (<1.0)
Bar/nightclub	1 (<1.0)
Other unspecified location	61 (1.8)
Unknown	35 (1.0)
**Total**	**3,429 (100)**

* Military status was unknown for 581 decedents.

^†^ Percentages might not total 100% due to rounding.

^§^ Ever served in the U.S. Armed Forces.

^¶^ Alaska, Arizona, Colorado, Connecticut, Georgia, Hawaii, Kansas, Kentucky, Maine, Maryland, Massachusetts, Michigan, Minnesota, New Hampshire, New Jersey, New Mexico, New York, North Carolina, Ohio, Oklahoma, Oregon, Rhode Island, South Carolina, Utah, Vermont, Virginia, and Wisconsin.

** Includes persons of any race.

#### Method and Location of Injury

The most common method used was a firearm (69.4%), followed by hanging/strangulation/suffocation (16.6%) and poisoning (9.2%) (Table 10). Most suicides among military personnel occurred at a house/apartment (77.2%), followed by a natural area (4.5%) and a motor vehicle (4.5%).

#### Toxicology Results of Decedent

Tests for alcohol were conducted for 47.3% of suicide decedents among former or current military personnel (Table 11). Results were positive for 37.8%; of these, 68.4% had BAC ≥0.08 g/dL. Tests for amphetamines, antidepressants, benzodiazepines, cocaine, marijuana, and opioids were conducted for 30.5%, 19.2%, 30.9%, 31.3%, 24.9%, and 34.1% of decedents, respectively. Of those tested for antidepressants, opioids, and benzodiazepines, results were positive for 37.6%, 26.0%, and 23.8%, respectively. Amphetamines, cocaine, and marijuana were detected in 5.3%, 3.1%, and 13.5% of decedents tested, respectively. When carbon monoxide was tested for (5.0%), results were positive for 42.4% of suicide decedents among former or current military personnel.

**TABLE 11:** Number* and percentage of suicide decedents among military personnel who were tested for alcohol and drugs whose results were positive,^†^ by toxicology variable — National Violent Death Reporting System, 27 states,^§^ 2015

Toxicology variable	Tested	Positive
	No. (%)	No. (%)
BAC^¶^	1,623 (47.3)	613 (37.8)
Alcohol <0.08 g/dL		167 (27.2)
Alcohol ≥0.08 g/dL		419 (68.4)
Alcohol positive, level unknown		27 (4.4)
Amphetamines	1,045 (30.5)	55 (5.3)
Anticonvulsants	508 (14.8)	78 (15.4)
Antidepressants	657 (19.2)	247 (37.6)
Antipsychotics	507 (14.8)	43 (8.5)
Barbiturates	844 (24.6)	25 (3.0)
Benzodiazepines	1,061 (30.9)	253 (23.8)
Carbon monoxide	172 (5.0)	73 (42.4)
Cocaine	1,072 (31.3)	33 (3.1)
Marijuana	855 (24.9)	115 (13.5)
Muscle relaxants	538 (15.7)	31 (5.8)
Opioids	1,168 (34.1)	304 (26.0)
Other drugs/substances**	593 (17.3)	425 (71.7)

**Abbreviation:** BAC = blood alcohol concentration.

* N = 3,429.

^†^ Percentage is of decedents tested for toxicology variable.

^§^ Alaska, Arizona, Colorado, Connecticut, Georgia, Hawaii, Kansas, Kentucky, Maine, Maryland, Massachusetts, Michigan, Minnesota, New Hampshire, New Jersey, New Mexico, New York, North Carolina, Ohio, Oklahoma, Oregon, Rhode Island, South Carolina, Utah, Vermont, Virginia, and Wisconsin.

^¶^ BAC ≥0.08 g/dL is over the legal limit in all states and is used as the standard for intoxication.

** Other drugs/substances indicated if any results were positive; levels for these drugs/substances are not measured.

#### Precipitating Circumstances

Precipitating circumstances were known for 91.6% of suicide decedents among former or current military personnel (Table 12). Of those with known circumstance information, 43.1% had a current diagnosed mental health problem and 37.1% were described as being depressed at the time of their death. Among those with a diagnosed mental health problem, depression/dysthymia (71.2%), posttraumatic stress disorder (18.6%), and anxiety disorder (12.8%) were the most common diagnoses. Approximately 27.5% of decedents had a history of mental health treatment and 21.0% were currently in treatment at time of death. Alcohol and other substance abuse problems were noted in 14.0% and 7.8% of decedents, respectively. With respect to interpersonal relationships, problems with an intimate partner (23.4%) were most frequently documented, followed by family relationship problems (6.5%). A physical health problem was the most common life stressor circumstance endorsed (37.2%), followed by a financial problem (8.2%), job problem (7.8%), and a recent criminal legal problem (7.1%). A substantial percentage of suicide decedents among military personnel (29.7%) reported a crisis during the preceding or upcoming 2 weeks. Approximately one third of decedents (34.7%) left a suicide note, 13.1% had made a previous suicide attempt, and 30.4% had a history of suicidal thoughts or plans. Approximately 23.2% had disclosed an intent to die by suicide; of those who disclosed their intent, the majority disclosed to a current or previous intimate partner (39.2%) or a family member (27.3%).

**TABLE 12:** Number* and percentage^†^ of suicides among military personnel,^§^ by precipitating circumstances — National Violent Death Reporting System, 27 states,^¶^ 2015

Precipitating circumstance	No. (%)
**Mental health/Substance abuse**	
Current diagnosed mental health problem**	1,354 (43.1)
Depression/dysthymia	964 (71.2)
PTSD	252 (18.6)
Anxiety disorder	173 (12.8)
Bipolar disorder	132 (9.7)
Schizophrenia	49 (3.6)
ADD/ADHD	10 (<1.0)
OCD	5 (<1.0)
Other	99 (7.3)
Unknown	94 (6.9)
Current depressed mood	1,166 (37.1)
History of ever being treated for a mental health problem	864 (27.5)
Current mental health treatment	660 (21.0)
Alcohol problem	441 (14.0)
Substance abuse problem (excludes alcohol)	244 (7.8)
Other addiction (e.g., gambling, sex)	19 (<1.0)
**Interpersonal**	
Intimate partner problem	735 (23.4)
Family relationship problem	204 (6.5)
Other death of family member or friend during past 5 years	225 (7.2)
Perpetrator of interpersonal violence during past month	75 (2.4)
Other relationship problem (nonintimate)	50 (1.6)
Suicide of family member or friend during past 5 years	50 (1.6)
Victim of interpersonal violence during past month	6 (<1.0)
**Life stressor**	
Crisis during previous or upcoming 2 weeks	934 (29.7)
Physical health problem	1,170 (37.2)
Argument or conflict	403 (12.8)
Job problem	244 (7.8)
Financial problem	257 (8.2)
Recent criminal legal problem	223 (7.1)
Noncriminal legal problem	98 (3.1)
Eviction or loss of home	113 (3.6)
School problem	8 (<1.0)
History of child abuse/neglect	25 (<1.0)
Physical fight (two people, not a brawl)	13 (<1.0)
Traumatic anniversary	19 (<1.0)
Exposure to disaster	16 (<1.0)
Caretaker abuse/neglect led to suicide	1 (<1.0)
**Crime and criminal activity**	
Precipitated by another crime	105 (3.3)
Crime in progress^††^	34 (32.4)
Terrorist attack	0 (0.0)
**Suicide event**	
Left a suicide note	1,091 (34.7)
History of suicidal thoughts or plans	956 (30.4)
History of suicide attempt(s)	411 (13.1)
**Suicide disclosure**	
Disclosed suicide intent	730 (23.2)
Disclosed intent to whom^§§^	
Previous or current intimate partner	286 (39.2)
Other family member	199 (27.3)
Friend/colleague	77 (10.5)
Health care worker	41 (5.6)
Neighbor	11 (1.5)
Other person	71 (9.7)
Unknown	45 (6.2)
**Total^¶¶^**	**3,141 (91.6)**

**Abbreviations:** ADD/ADHD = attention deficit disorder/attention deficit hyperactivity disorder; OCD = obsessive-compulsive disorder; PTSD = posttraumatic stress disorder.

* Includes suicides with one or more precipitating circumstances. More than one circumstance could have been present per decedent.

^†^ Percentages might not total 100% due to rounding.

^§^ Ever served in the U.S. Armed Forces.

^¶^ Alaska, Arizona, Colorado, Connecticut, Georgia, Hawaii, Kansas, Kentucky, Maine, Maryland, Massachusetts, Michigan, Minnesota, New Hampshire, New Jersey, New Mexico, New York, North Carolina, Ohio, Oklahoma, Oregon, Rhode Island, South Carolina, Utah, Vermont, Virginia, and Wisconsin.

** Includes decedents with one or more current diagnosed mental health problems. Therefore, sums of percentages for the diagnosed conditions exceed 100%. Denominator in percentages is the number of decedents with one or more current diagnosed mental health problems.

^††^ Denominator is decedents involved in an incident that was precipitated by another crime.

^§§^ Denominator is decedents who disclosed intent.

^¶¶^ N = 3,429. Circumstances were unknown for 288 decedents.

## Discussion

Violent deaths occur among males and females of all ages, races, and ethnicities. NVDRS data help identify populations particularly affected by violence-related injuries leading to death. Violence also occurs in many forms. NVDRS not only provides details on specific manners of violent deaths, but also has the capacity to identify common risk factors for multiple forms of violence. These details can increase knowledge about the circumstances associated with violence and can help public health authorities develop and guide data-informed, effective approaches to violence prevention. The occurrence of violent deaths also varies greatly across states (*1*). Recent expansion of NVDRS in 2014 and 2016 increased the system’s operation to 40 states, the District of Columbia, and Puerto Rico. With these expansions, NVDRS provides more comprehensive violent death information and equips more states and communities with data for appropriate public health action at the local level. The 27 states that provided data for this report accounted for 45.5% of violent deaths and represented half (46.9%) of the U.S. population in 2015 (*1*). Further expansion of NVDRS to the remaining states and territories will allow CDC to provide valuable information on the scope of violent deaths in the United States and to guide development of violence prevention efforts at the national level.

Violence is preventable and reducing violent deaths in communities is possible with evidence-based approaches (*10*). CDC released a series of technical packages to assist communities and states in identifying approaches and strategies with the best available evidence to prevent violence (*10*). The five technical packages include strategies; approaches; and specific programs, practices, and policies with evidence of effects on risk for child abuse and neglect, IPV, youth violence, sexual violence, and suicide. Each package considers the multifaceted and interactive effects of individual, relationship, family, school, and community factors that can influence violence-related outcomes by identifying strategies and approaches that are representative of different levels of the social ecology. The strategies and approaches also are intended to work in combination and reinforce each other in a comprehensive and long-term way. A number of sectors are instrumental in the implementation of these packages, including education, government, social services, justice, housing, businesses, and faith-based organizations (*10*).

The findings in this report indicate that demographic variations continue to exist in the manner of death from violence-related injuries. The majority of violent deaths are suicides, comprising 65.1% of deaths collected in NVDRS. Suicides occurred at higher rates among non-Hispanic American Indian/Alaska Natives, followed by non-Hispanic whites, and rates were highest among persons aged 45–64 years. Homicide rates were highest among adults aged 20–29 years, especially men. For both males and females, rates of homicide were highest among non-Hispanic blacks and American Indian/Alaska Natives. Among males, the homicide rate among blacks was 35.0 per 100,000 population, a rate approximately two times that for non-Hispanic American Indian/Alaska Native and 12 times that for non-Hispanic white males. A firearm was the most common method used in homicides and suicides, and most deaths of undetermined intent were poisonings.

The racial/ethnic differences in homicide rates identified in this report, particularly among youths and young adult males, warrants prioritizing race/ethnicity-related disparities in violence prevention. Racial/ethnic minority youths often live in communities with concentrated poverty, stressed economies, residential instability and neighborhood disorganization, access to firearms and illegal drugs, and low community cohesion and informal controls. All these conditions are associated with violence and violence-related injuries (*11*). Prevention efforts will achieve greater population-level reductions in violence when salient neighborhood and community-level contributors to violence are targeted (*12*). Evaluations of programs such as Baltimore’s *Safe Streets,* Crime Prevention Through Environmental Design (CPTED), business improvement districts, and policies such as the earned income tax credit (*11*) have confirmed the value in employing these types of community-level strategies in reducing violence. Evidence also suggests that these strategies and other universal policies that focus on general community improvements can have substantial impact on decreasing the racial/ethnic gap in violence (*13*).

NVDRS data provide important insights into circumstances for each manner of death. Current diagnosed mental health problems, intimate partner problems, and recent crises were frequent precipitants of suicide. In addition, 31.9% of suicide decedents had a history of suicidal thoughts or plans and 23.5% had disclosed their suicide intent. These precipitants are well documented as important risk factors to target in suicide prevention (*14*). Despite the high frequency of reported mental health problems and suicidal intent among decedents, less than one third were known to be receiving treatment at the time of death.

CDC’s suicide prevention technical package (*15*) contains the following seven strategies for reducing suicide and suicidal behaviors: 1) strengthen economic supports, 2) strengthen access and delivery of suicide care, 3) create protective environments, 4) promote connectedness, 5) teach coping and problem-solving skills, 6) identify and support people at risk, and 7) lessen harms and prevent future risk. Each includes examples of specific approaches that states and communities can implement to advance the strategy. On the basis of this report’s findings, several approaches, including social-emotional learning programs, treatment for people at risk for suicide and treatment to prevent reattempts, enhancing parenting skills and family relationships, and others will be important to include when developing suicide prevention programs. Suicide prevention efforts are best achieved if these approaches and strategies are used in combination and reinforce each other to reduce risk for suicide, as well as to have cross-cutting impact on other forms of violence (*15*). By using NVDRS data, suicide prevention experts can guide planning and implementation and track outcomes of suicide prevention strategies and approaches within their states and communities. The strategies in the technical package support the goals and objectives of the National Strategy for Suicide Prevention (NSSP) (*16*) and the National Action Alliance for Suicide Prevention’s priority to strengthen community-based prevention (*17*).

NVDRS homicide circumstance data indicate that homicide decedents were most often males killed as the result of an argument or conflict or during the commission of a crime (predominately assault/homicide). In contrast, among females almost half of homicides were related to IPV; a current or former spouse/intimate partner was identified as the perpetrator in 51.4% of homicides with known perpetrators. These findings were similar to a recent NVDRS report that highlighted the differential impact of IPV-related homicides among young and racial/ethnic minority women (*18*).

Efforts to reduce IPV among women include screening women of childbearing age for IPV and referring those who have a positive screen to intervention services (*19*) and providing counseling services for pregnant women (*20*). Screening that is conducted in a culturally sensitive way is important to minimize any threats to safety. Strategies also include providing support to survivors, empowering bystanders, engaging men and boys as allies (*21*,*22*), and teaching youths about safe and healthy relationships before they begin dating (*21*,*23*,*24*). These prevention strategies also can benefit from cross-cutting efforts that incorporate changing social norms, including harmful gender norms that condone violence, and societal conditions that serve to maintain harmful norms and inequality across sex, racial/ethnic, and income groups. Evidence for the effectiveness of these and other approaches is outlined in CDC’s IPV prevention technical package (*21*).

NVDRS data underscore that suicides and homicides frequently are preceded by relationship problems. These findings highlight that many forms of violence are interconnected and might share the same root causes (*25*). Therefore, violence prevention and intervention efforts can be broadened to address multiple forms of violence and increase their impact (*25*). This report’s findings also support the need to implement programs that develop social and emotional skills (e.g., problem solving, conflict resolution, and individual coping skills) and cultivate supportive relationships to protect against violent injuries and death. For example, the Safe Dates Program, a school-based program designed to reduce dating violence among adolescents, has shown promise for reducing long-term physical and sexual dating violence as well as peer violence victimization and weapon carrying (*21*,*23*,*24*). Furthermore, primary prevention strategies designed to teach skills that reduce aggressive behavior toward others and improve social skills, emotional well-being, and self-esteem can be targeted toward preadolescents and early adolescents before violent behaviors and patterns begin (*11*). CDC’s youth violence technical package emphasizes the preventive effects of skill development programs for youths and prevention approaches that address relationships and influence school and community environments (*11*).

Substance use is another frequent precipitant of suicide and interpersonal violent behavior. Toxicology results documented a high prevalence of alcohol, especially with BAC ≥0.08g/dL (over the legal limit) among suicide and homicide decedents tested for substance use. Alcohol use is a robust predictor of suicidal behavior (*26*) and victimization (*27*). Intoxication can lead to disinhibition, enhance feelings of hopelessness and depression, and impair judgment that can lead to impulsive behaviors (*14*). Alcohol use can also reduce awareness and perception of surrounding risks, thus increasing one’s vulnerability to being victimized (*28*). Opioids (illicit or prescription) were the most common substances detected in deaths of undetermined intent. Whether these deaths were the result of unintentional drug poisonings (which have increased substantially in recent years) (*29*) or suicides is unknown. Unintentional opioid overdose has been recognized as an epidemic (*29*). CDC issued the Guideline for Prescribing Opioids for Chronic Pain to help address the epidemic; support safer prescribing practices; and reduce opioid misuse, opioid use disorder, and overdose (*30*).

NVDRS collects more complete information than other data sources on legal intervention deaths (*31*) and unintentional firearm deaths (*32*). Findings from 27 states indicate that the largest proportion of deaths due to legal intervention were among non-Hispanic white males; however, the rate among non-Hispanic black males was approximately two times that of their white male counterparts. These findings are consistent with a published study (*33*), but further analyses are needed. Increased understanding about the magnitude and circumstances of these deaths will be essential to developing appropriate prevention strategies and monitoring their effectiveness. NVDRS also has been recognized as a reliable source of data on unintentional firearm deaths (*32*) and its capability to provide details about victims and shooters (*34*). This report indicated that 50.5% of unintentional firearm deaths were self-inflicted, but approximately 41% were known to be inflicted by another person. Most of these deaths occurred while playing with a gun, accidentally pulling the trigger, or while thinking the gun was unloaded, highlighting the importance of safe storage practices and education about safe handling of firearms.

NVDRS data permit examination of violent deaths involving specific populations. Findings in this report indicate that most decedents of suicide among former or current military personnel were non-Hispanic white males aged 45–74 years. The most common precipitating circumstances associated with suicide among military personnel were similar to those among all male suicide decedents (i.e., having a depressed mood, a history of suicidal thoughts and plans, and experiencing intimate partner problems or a crises near the time of death).

NVDRS data have been used to examine the circumstances surrounding suicides among veterans and active duty personnel and develop suicide prevention programs (*35*–*37*). For example, the Virginia Violent Death Reporting System (VVDRS) found that suicide rates in Virginia during 2003–2010 were higher among active duty members than civilians or veterans (*35*). Although a lesser percentage of active duty members exhibited warning signs, such as disclosing intent or prior suicide attempts, active duty suicide decedents had a number of factors that were described as potentially increasing their suicide risk, including intimate partner conflict, potential for a stressful job, and easy access to methods of fatal injury. The VVDRS report recommended focusing more on these identified warning signs as opportunities for prevention and intervention (e.g., intimate partner conflict, criminal legal problems, and other life crises) while continuing to encourage service members to talk to someone if they have suicidal thoughts (*35*). 

Several states participating in NVDRS are working also with Veterans Affairs offices in their respective states to use the data for prevention efforts to reduce suicides among veterans and active duty personnel. The Oregon Violent Death Reporting System (ORVDRS) found that the rate of death by suicide among veterans in Oregon increased during 2001–2012, and the suicide rate was substantially higher among veterans than among nonveterans, particularly among young male veterans (*36*). In 2013, Oregon’s Department of Veterans’ Affairs convened a 10-member interagency team to attend a policy academy sponsored by the Substance Abuse and Mental Health Services Administration’s Technical Assistance Center for supporting service members, veterans, and their families. ORVDRS staff participate in Oregon’s Service Members, Veterans and Their Families Workgroup, which identified suicide prevention as a strategic priority. The workgroup coordinates information and activities across public agencies and sectors of Oregon’s business and nonprofit communities.

NVDRS has notable utility for defining public health priorities, developing and evaluating programs and policies, and conducting research regarding violent deaths (*37*). For example, in 2015 the Arizona Violent Death Reporting System (AZVDRS) found high suicide rates among adults aged ≥65 years in the state. Suicide rates per 100,000 population were higher among men than women (39.2 versus 6.0), non-Hispanic whites than Hispanic and other races (25.4 versus 2.8), veterans than nonveterans (48.7 versus 12.7), and divorced than married adults (41.6 versus 15.4). Approximately 80% of suicides among older adults involved the use of a firearm. The AZVDRS report recommended that primary care providers implement routine standard screening for suicidal ideation and related issues (e.g., physical symptoms, sleep problems, and depression) among older adult patients. The AZVDRS report also recommended reducing access to lethal means among older adults who have depression or suicidal ideation (*37*).

NVDRS is also relevant to two national prevention initiatives, NSSP and *Healthy People 2020* (*16*,*38*). NSSP is a comprehensive national agenda for suicide prevention (*16*). In particular, NVDRS is relevant to NSSP goals of increasing the timeliness and usefulness of surveillance systems related to suicide prevention and evaluating the outcome and effectiveness of suicide prevention interventions. *Healthy People 2020* includes objectives for reducing the number of suicides, homicides, and firearm-related deaths and increasing the number of states that link data on violent deaths from death certificates, law enforcement reports, and coroner/medical examiner reports at state and local levels (*38*). NVDRS data can be used to measure states’ progress toward these goals by allowing for the examination of changing patterns in circumstances and risk profiles, which is not possible with other data sources.

## Limitations

The findings provided in this report are subject to at least eight limitations. First, NVDRS data are available from a limited number of states and therefore are not nationally representative. Second, the availability, completeness, and timeliness of data are dependent on partnerships among state Violent Death Reporting System programs and state health departments, vital statistics registrars’ offices, coroners/medical examiners, and law enforcement personnel. Data sharing and communication among partners is particularly challenging when states have independent county coroner systems rather than a centralized coroner/medical examiner system, a large number of law enforcement jurisdictions, or both. NVDRS incident data might be limited or incomplete for areas in which these data-sharing relationships are not developed fully. Third, toxicology data are not collected consistently across all states or for all alcohol and drug categories. Toxicology testing is not conducted for all decedents, so the percentages of those with positive results for specific substances might be affected by selective testing patterns in coroner/medical examiner offices (*39*). Fourth, abstractors are limited to the data included in the investigative reports they receive. Reports might not fully reflect all information known about an incident, particularly for homicides and legal intervention deaths, when data are less readily available until after a full investigation and adjudication are completed. Fifth, case definitions present challenges when a single death is classified differently in different documents (e.g., unintentional in a law enforcement report, homicide in a coroner/medical examiner report, and undetermined on the death certificate). NVDRS abstractors reconcile these discrepancies using standard NVDRS case definitions and select a single manner of death on the basis of all source documents; the manner of death assigned must be consistent with the manner of death noted in at least one source document. Sixth, variations in coding might occur depending on the abstractor’s level of experience. For this reason, CDC provides abstractor training and states conduct blinded reabstractions of cases to test consistency and identify training needs. Seventh, medical and mental health information (e.g., type of condition and whether the decedent was currently receiving treatment) often are not captured directly from medical records but from coroner/medical examiner reports and the decedent’s family members and friends. Therefore, the completeness and accuracy of this information is limited by the knowledge of the informant. Finally, protective factor data (i.e., characteristics or circumstances that reduce the risk for violent death) are not collected by NVDRS because of the nature of death certificates, coroner/medical examiner reports, and law enforcement reports, which typically contain only circumstances associated with risk factors.

## Conclusion

Public health surveillance is the foundation for public health practice. Surveillance is essential to monitoring the prevalence and incidence of violence-related fatal injuries, defining priorities, and directing programmatic and violence prevention activities (*40*). Plans are under way to expand NVDRS to 50 states, moving toward achieving the ultimate goal of providing nationally representative data by including all states, U.S. territories, and the District of Columbia. This expansion will not only make violent death information available for every state to inform local prevention efforts, but will allow for the system’s capacity to measure the need for and effects of violence prevention policies, programs, and practices at the national level.

## Footnotes

* The name of the Institute of Medicine was changed to the National Academy of Medicine, effective July 1, 2015.

† To be included in NVDRS, deaths of undetermined intent must have some evidence of the possibility that the intent was purposeful, including use of a weapon or other evidence that force was used to inflict the injury. Most commonly, the coroner/medical examiner is unsure whether the death was a suicide or unintentional.

§ A total of 42 states and territories participate in NVDRS. California began collecting data in 2005 but ended data collection in 2009. In 2017, California resumed data collection.

¶ Frequencies and rates of violent deaths included in this report will differ slightly from the frequencies and rates of violent deaths reported by NVDRS WISQARS, which excludes nonresident deaths that occur in participating states (i.e., occurrent deaths). NVDRS tracks both resident and occurrent violent deaths in the overall data set, and the numbers in this report reflect both. States are expected to collect information on violent deaths among their residents, wherever they occur, and fatal violent injuries occurring within their borders irrespective of the decedent’s residence status. If the states of residence and injury occurrence are both participating NVDRS states, the state of injury occurrence is responsible for collecting the information. By making this differentiation of responsibility, duplicate reporting is avoided.

** Drug trade is defined as the buying, selling, or passing of drugs in exchange for goods or money. Drug involvement includes drug use in addition to drug trade.
